# Mapping the Shapes of Phylogenetic Trees from Human and Zoonotic RNA Viruses

**DOI:** 10.1371/journal.pone.0078122

**Published:** 2013-11-01

**Authors:** Art F. Y. Poon, Lorne W. Walker, Heather Murray, Rosemary M. McCloskey, P. Richard Harrigan, Richard H. Liang

**Affiliations:** 1 British Columbia Centre for Excellence in HIV/AIDS, Vancouver, British Columbia, Canada; 2 Department of Medicine, University of British Columbia, Vancouver, British Columbia, Canada; 3 School of Medicine, University of California San Diego, La Jolla, California, United States of America; George Washington University, United States of America

## Abstract

A phylogeny is a tree-based model of common ancestry that is an indispensable tool for studying biological variation. Phylogenies play a special role in the study of rapidly evolving populations such as viruses, where the proliferation of lineages is constantly being shaped by the mode of virus transmission, by adaptation to immune systems, and by patterns of human migration and contact. These processes may leave an imprint on the shapes of virus phylogenies that can be extracted for comparative study; however, tree shapes are intrinsically difficult to quantify. Here we present a comprehensive study of phylogenies reconstructed from 38 different RNA viruses from 12 taxonomic families that are associated with human pathologies. To accomplish this, we have developed a new procedure for studying phylogenetic tree shapes based on the ‘kernel trick’, a technique that maps complex objects into a statistically convenient space. We show that our kernel method outperforms nine different tree balance statistics at correctly classifying phylogenies that were simulated under different evolutionary scenarios. Using the kernel method, we observe patterns in the distribution of RNA virus phylogenies in this space that reflect modes of transmission and pathogenesis. For example, viruses that can establish persistent chronic infections (such as HIV and hepatitis C virus) form a distinct cluster. Although the visibly ‘star-like’ shape characteristic of trees from these viruses has been well-documented, we show that established methods for quantifying tree shape fail to distinguish these trees from those of other viruses. The kernel approach presented here potentially represents an important new tool for characterizing the evolution and epidemiology of RNA viruses.

## Introduction

**Figure 1:**
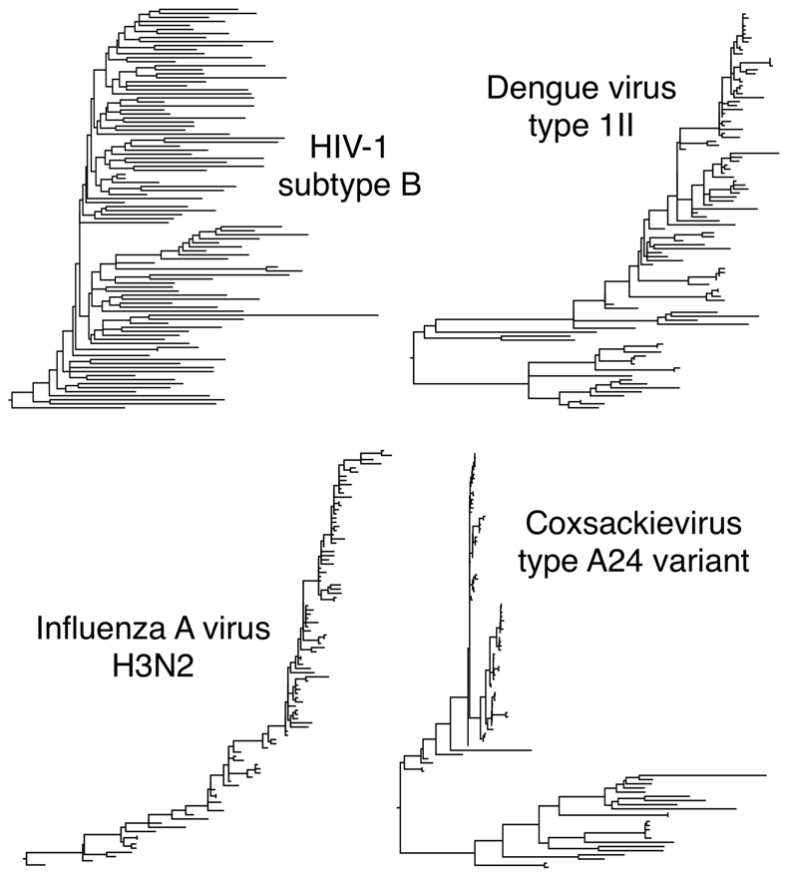
Diversity in phylogenetic tree shapes for animal RNA viruses. These phylogenies were generated from samples of genetic sequences from HIV-1 subtype B (HIV1-B), dengue virus serotype 1II (DEN-1II), influenza A virus serotype H3N2 (IAV-H3), and coxsackievirus A24 variant (CA24v).

Trees have long been used as a metaphor for making sense of a diverse living world. The modern phylogenetic tree is a model of the evolutionary relatedness of populations or individuals, which is increasingly based on the similarity of their genetic makeup. Phylogenies have become an indispensable tool for the study of biological variation by providing a framework for modelling evolutionary processes along lineages of descent from common ancestors. In particular, phylogenies are used extensively in the study of viruses. Viruses, especially those with RNA genomes, exhibit some of the fastest rates of molecular evolution in the natural world, driven by high rates of mutation [1], large population sizes and short generation times. Many viral lineages can proliferate on time scales measured in days, as opposed to the millions of years it would require for many branches on the macroscopic ‘Tree of Life’ to grow to the same extent. The substantial variation in the shapes of RNA virus phylogenies (Figure 1) may be determined by epidemiological processes that operate at the scale of the host population. These processes may in turn be shaped by the ongoing evolution of the virus. The study of this multi-level interaction is known as ‘phylodynamics’ [2].

Identifying the reproducible shape characteristics among virus phylogenies and the epidemiological and evolutionary determinants of these shapes are key research questions of phylodynamics [3]. For instance, the stark contrast between phylogenies generated respectively from influenza A virus (IAV) and human immunodeficiency virus (HIV) type 1 sequences is a prototypical example of how virus phylogenies can be shaped by phylodynamic processes [4], [5]. A phylogeny reconstructed from sequences of IAV hemagglutinin, a receptor-binding glycoprotein which is a primary target of the host immune system, has a distinctively pectinate (‘comb-like’) shape (Figure 1) that is hypothesized to be driven by the short infectious period and antigenic drift of IAV, in which very few lineages persist between successive epidemics [6]. In contrast, the life-long nature of HIV infection and ongoing transmission among hosts has enabled rapidly diverging lineages to proliferate and persist over time, leading to ‘star-like’ phylogenies with short branches near the root and long branches at the tips (Figure 1).

Until recently, our understanding of phylodynamics through the comparative study of virus phylogenies has been generally limited to conspicuous differences in tree shape, such as that between HIV and IAV phylogenies, because tree shapes are inherently difficult to quantify. A phylogenetic tree is an acyclic graph in which the nodes represent either observed entities (leaf nodes) or their common ancestors (internal nodes) which must be reconstructed from the variation observed at the leaf nodes. These nodes are linked by branches whose lengths represent either the passage of time or the amount of evolutionary change that has occurred between nodes. As a result, the shape of a phylogenetic tree is a complex object that incorporates both the lengths and distribution of branches among nodes. There is an abundance of descriptive statistics of tree shape that focus on the concept of ‘tree balance’, which refers to the skewed distribution of internal nodes in a tree due to variation in rates of branching [7]. For example, the Sackin index [8] counts the number of internal nodes along the path from each leaf node to the root of the tree. When branching events occur preferentially along specific lineages, the corresponding phylogeny should tend to have a higher Sackin index. However, tree balance statistics are notoriously sensitive to sample size [9] and capture only one abstraction of tree shape; for example, differences in branch lengths are generally not taken into consideration.

Here, we present a new approach to the study of phylodynamics using a versatile method from machine learning [10]. We begin with a function 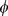 that maps an object 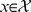 into a representation space 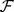 that is more amenable to analysis than 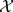. A well-conceived 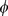 can be extremely useful for dealing with structured data such as trees, which otherwise have no direct numerical representation. For example, Sackin’s index is a function that maps from the space of all possible tree shapes to the set of all positive integers. While integer representations of tree shape are far more convenient for analysis, this choice for 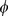 is not adequate for our purposes because it captures only a small fraction of the variation among tree shapes. When dealing with complex data, it is more appropriate to use a function that maps objects into a representation space with many dimensions. For example, one could devise a function to be applied to blocks of text that counted the occurrence of substrings of any length. Every possible substring adds a new dimension to the feature space, as 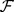 is referred to in the field of pattern recognition. As the data become larger and more complex, the number of possible features (and thereby the dimensionality of 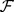) may become enormous. Thus, while 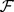 can provide a detailed representation of complex objects, it also becomes infeasible to operate within. Because we are interested specifically in finding linear relations between objects that have been mapped to 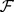, we bypass the difficult task of explicitly mapping each object to 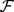 by defining a function that efficiently evaluates the inner product between two representations of objects in 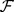: 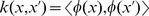 where 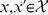. This procedure is known as the *kernel trick* and 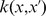 is referred to as the kernel function [11]. As kernel functions are generally designed to take larger values when 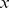 and 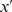 have a greater number of features in common, they can each be interpreted as a non-linear measure of similarity.

To provide an intuition to this use of the kernel trick, imagine a person who has encountered three texts written in English – specifically, the texts contained in Agapow and Purvis [12], Huelsenbeck *et al.* [13], and Robinson and Foulds [14]. Moreover, suppose this person has absolutely no knowledge of the English language, let alone phylogenetics. Realizing that there are discrete shapes (words) and that many of them appear repeatedly, he or she carries out the lengthy task of cataloguing the number of times that each shape appears in each text. (For the sake of example, we ignore such complications as punctuation and typography.) While there are many shapes that are unique to a given text, there is a much smaller subset of shapes that appear in two or more texts. Therefore, in order to compare two of these lists, it is convenient to multiply the counts of shapes that appear in both lists (knowing that unique shapes would multiply to zero) and sum these products to generate a similarity score. Multiplying the counts has the useful effect of emphasizing shapes that appear in both texts at roughly equal frequencies. For example, ‘tree’ appears 37 times in both [12] and [14], which contributes a score of 1369. In contrast, if all but one of the 74 occurrences of ‘tree’ appeared in one of the two texts, then the score would have increased by only 73. This exercise yields three numbers corresponding to the pairwise comparisons: 264630 [12], [13], 141470 [12], [14], and 277008 [13], [14]. However, [13] is a much lengthier text that the other two, which has inflated the scores for pairwise comparisons that included this text. It is necessary to normalize each score for the size of the corresponding texts [15]; upon doing so, our imagined person is able to conclude that [12] and [14] are the most similar (0.84) without knowing that these papers are, in fact, both on the subject of phylogenetic tree shape, whereas [13] (with normalized scores of 0.82 and 0.78, respectively) concerns the problem of inferring the root of a phylogeny.

**Figure 2:**
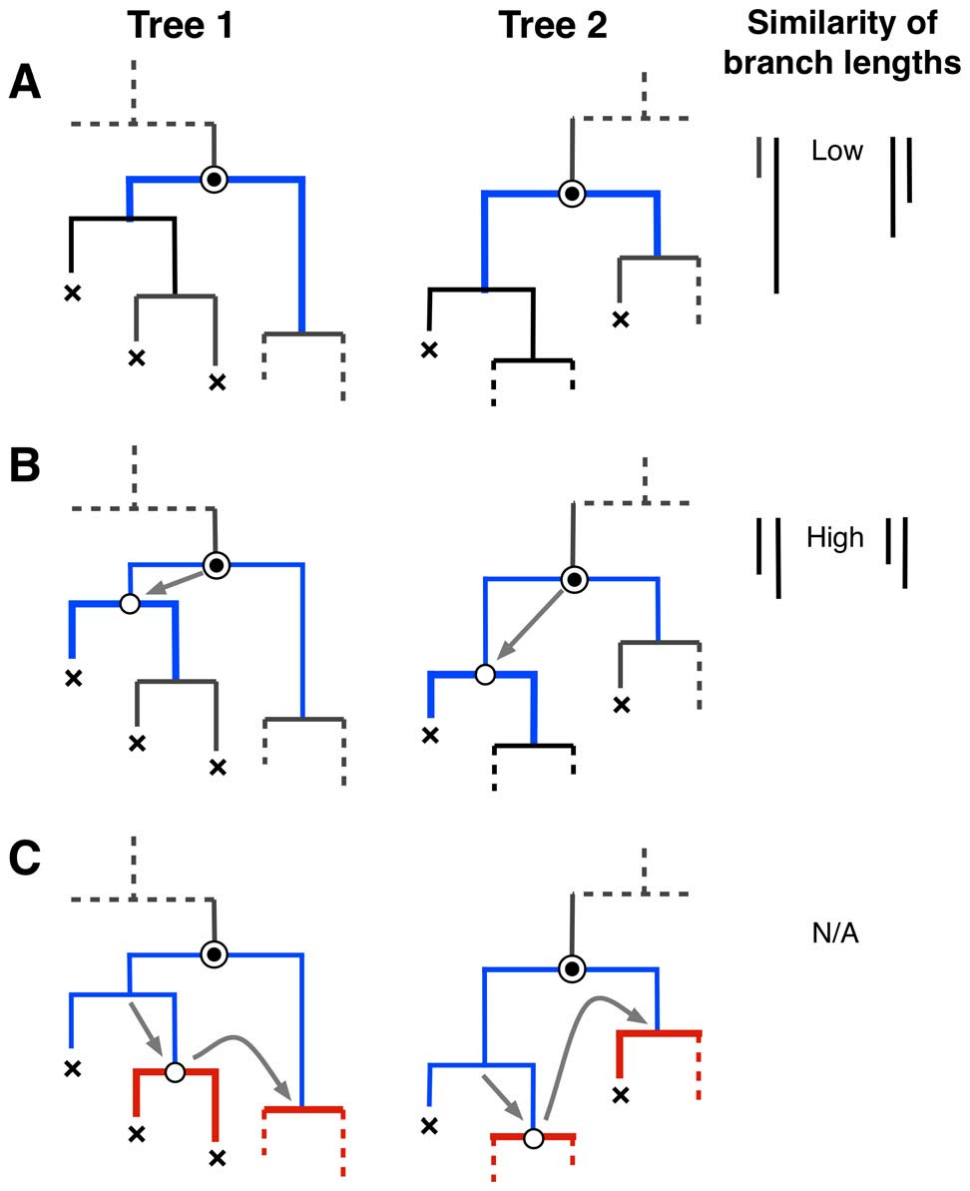
Kernel-assisted comparison of two tree shapes. For trees comprising 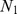 and 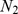 nodes, respectively, there are 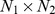 pairs of nodes to evaluate. (A) Starting from a given pair of nodes (indicated in figure by circles with double-outlines), the algorithm finds the largest common subset tree rooted at these nodes. First, we find that for both nodes, neither of the branches terminate at a ‘leaf node’ (marked with ‘
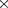
‘). This match contributes a relatively small amount to our kernel score, not only because the matching subset trees (highlighted in thick blue lines) comprise only one node each, but also because their discordant branch lengths lead to a substantial penalty. (B) Next, we descend down the left branch in both trees. The current nodes (open circles) in both trees spawn one leaf node and one internal node; therefore, the subset trees continue to match. In addition, their branch lengths are similar, so their contribution to the cumulative kernel score is given greater weight. (C) Finally, we descend down the right branch in both trees and find that the subset trees no longer match beyond this point. We also proceed down the right branch of the reference nodes and find no match, so our traversal of the two trees from these nodes is complete and we restart our search at the next pair of nodes.

To construct a kernel function on phylogenetic tree shapes, we adapted a natural language processing kernel function [16] that was originally designed to classify text on the basis of its syntactic structure (a generative tree in which words descend from linguistic precursors [15]). Our modified kernel function 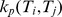 extracts all the subset trees that are the common features of two phylogenetic trees 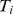 and 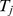 (Figure 2). A subset tree is a contiguous collection of descendants of a specific node, but unlike a subtree, it does not necessarily include all of the descendants. Thus, our approach is similar to the Robinson-Foulds metric [14] which compares alternative trees for a given set of taxa by counting the number of subtrees in common. Unlike the Robinson-Foulds metric, however, our kernel function not only allows us to compare trees relating different numbers and kinds of taxa, but it also accounts for differences in the branch lengths between matching subset trees. We assess the performance of our kernel function against nine measures of tree shape at classifying simulated phylogenies with varying rates of speciation, and then apply our function to a large collection of human and zoonotic RNA virus phylogenies.

## Methods

### Data Collection

A working list of RNA viruses associated with human pathologies was obtained from the ViralZone web resource (http://viralzone.expasy.org/all_by_species/678.html) [17]. For each virus, we attempted to gather as many sequences as available covering a region of the genome with extensive genetic variation, based on prior knowledge or phylogenetic studies in the literature. When available, we used curated virus-specific sequence databases. HIV-1 *nef* gene sequences were obtained from the Los Alamos National Laboratory (LANL) HIV Sequence database (http://www.hiv.lanl.gov/) using subtype-specific search queries excluding sequences annotated as recombinants, and restricting the result to one sequence per patient. Further screening for HIV-1 recombinant sequences was carried out using SCUEAL (Subtype Classification Using Evolutionary ALgorithms) [18]. Hepatitis C virus E1 sequences were obtained from the LANL HCV sequence database (http://hcv.lanl.gov) [19] using genotype-specific search queries excluding recombinants. Search results were reduced to one sequence per patient based on patient clone annotation in the sequence headers using a custom Python script, as this functionality was not available for the LANL HCV sequence database. Influenza A virus hemagglutinin (HA) sequences were obtained from the National Institute of Allergy and Infectious Diseases (NIAID) Influenza Research Database (http://www.fludb.org), using subtype-specific search queries excluding isolates from non-human hosts and laboratory strains.

Gene sequences from all other viruses were obtained from the GenBank sequence database by primary organism taxonomy identifier (‘txid’). Queries yielding fewer than 100 records irrespective of gene were omitted at this stage. In all cases, sequence data were downloaded as FASTA formatted files; these published data were already anonymized and did not include any information about patients’ medical records. Sequences annotated as multiple clonal isolates from the same patient, sequences from patents or laboratory strains were excluded from these data. For all viruses other than HCV, HIV and IAV, we retrieved collection dates and host organism data, when available, for each sequence by its Genbank accession number using an automated query algorithm implemented in Python. By convention, Genbank records are annotated with this information in a ‘source’ field; however, this field is not always included in data submissions. For HCV, HIV and IAV sequences, which were already screened for human hosts, collection dates were parsed from sequence headers that were generated by the respective database engines.

### Sequence Alignment

For each FASTA file, one sequence was selected as a reference for initial pairwise alignment of all other sequences using an implementation of the modified Gotoh algorithm in HyPhy [20], [21]. Sequences were trimmed to the aligned portion and converted to a multiple sequence alignment using MUSCLE [22] version 3.8 under default settings. All alignments were visually inspected and adjusted using Se-Al (Andrew Rambaut, http://tree.bio.ed.ac.uk/software/seal). Columns in the alignment comprising over 50% gaps were excluded. A table of all viruses, subtypes and genomic regions used for phylogenetic reconstruction is provided as Supporting Information (Table S1).

Initial phylogenies were generated using FastTree (version 2.1.4) [23] under the general time-reversible model of nucleotide substitution. These phylogenies were used to identify clusters of sequences that could represent multiple isolates from the same individual, which we determined by evaluating the publications linked with sequences within putative clusters. Furthermore, the initial phylogenies were used to partition or filter alignments by genotype or subtype, because the Genbank sequence records were not consistently annotated with this information. The data sets that were partitioned or filtered using the initial phylogenies were: astrovirus, dengue virus type 1, hepatitis delta virus, hepatitis E virus, influenza A virus H1, Japanese encephalitis virus, Norwalk virus genotype II, poliovirus (wild and vaccine variants), rabies virus, rotavirus A, rubella virus, sapovirus, tick-borne encephalitis virus, and West Nile virus.

### Phylogenetic Reconstruction

Although it is possible to apply our kernel method (described below) to an unrooted tree by counting subtrees from the deepest node outwards, phylodynamic studies of tree shapes have conventionally been carried out on rooted trees which can be more informative about evolutionary processes. The root of a phylogeny is a hypothesis on the location of most recent common ancestor (the earliest point in time) on the tree. We used an outgroup criterion [13] to infer rooted phylogenies from the alignments. This method relies on selecting an outgroup sequence that is sufficiently closely related to the true ancestor. For each alignment, we selected an outgroup sequence based on previous phylogenetic studies on the respective viruses in the literature, either a representative sequence from a closely-related virus genotype or subtype (*e.g.*, HIV-1 subtype D), or the earliest/prototype virus isolate (*e.g.*, West Nile virus isolate B956 [24]). A list of outgroup sequence accession numbers and literature citations is provided in Table S1. We assessed the suitability of the respective outgroup sequences by generating preliminary rooted trees using FastTree2. While results based on tree balance statistics were sensitive to outgroup rooting, our results based on the kernel analysis were robust to choice of outgroup (data not shown).

For alignments with 200 or more sequences, we generated 100 replicate subsets of 100 sequences sampled at random without replacement from each data set for phylogenetic reconstructions. This resampling controlled for the effect of excessive variation in tree size (number of sequences) on comparisons of tree shape. Otherwise, a single phylogeny was generated for each alignment with fewer than 200 sequences (Crimean-Congo hemorrhagic fever virus; Chikungunya virus; encephalomyocarditis virus; HIV-1 subtype A; Hantaan virus; human rotavirus C; influenza A virus H2; influenza C virus; Murray valley encephalitis virus; mumps virus; Oropouche virus; human parainfluenza virus; Rift valley fever virus; rhinovirus A; rotavirus A genotypes 3, 4, and 12; rubella virus clade 2; sapovirus; tick-borne encephalitis virus, European and Far-Eastern strains; yellow fever virus). For data sets in which 200 or more sequences were annotated with collection dates, we generated 10 additional samples of 100 sequences drawn uniformly at random with respect to the year of collection. Phylogenies reconstructed from these latter samples provided a means to assess the sensitivity of our analyses to biased sample collection with respect to time.

Phylogenetic reconstruction for every sample was carried out using a multithreaded implementation of RAxML (version 7.3.0) [25] under a general time-reversible model of nucleotide substitution with 25 rate categories across sites, followed by optimization under a discretized gamma distribution of rate categories (GTRCAT). All phylogenies contained a number of ‘soft’ polytomies, in which the phylogenetic reconstruction was unable to assign a non-zero branch length at an internal node due to inadequate genetic divergence. Because RAxML is limited to strictly bifurcating representations of phylogenetic trees (with the exception of the deepest node from which all branches are interpreted as descendants), soft polytomies were automatically resolved by the software into an arbitrary binary tree with branch lengths set to a small non-zero value. By randomizing the resolution of soft polytomies into binary trees, we found that our results were robust to this source of uncertainty in phylogenetic reconstruction (data not shown).

For every phylogeny, we calculated two popular summary statistics of tree balance, Sackin’s index and Colless’ index following the formulae in [26]. Next, we generated a kernel matrix for all phylogenies using an implementation of our phylogenetic tree shape kernel as described in the following section.

### Kernel Analysis

To apply the kernel function to these data, we needed to iterate through all internal nodes in two phylogenies and count all subset trees with the same topology (branching order) in both phylogenies (Figure 2). This is akin to quantifying the similarity of two manuscripts by counting the number of times that various words appeared in both texts, as illustrated by our example in the Introduction. Unlike a text, however, the shape of a tree is an ambiguous characteristic of a phylogeny. Rotating the branches around a node can produce a tree that has a different shape, despite remaining evolutionarily equivalent to the original. The number of different shapes for a tree of 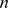 tips (where we distinguish left from right and, for the moment, ignore branch lengths) is given by the Catalan numbers 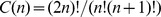. For example, a tree of 100 tips has nearly 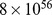 shapes. This presents a problem: how do we know which resolutions of shapes are being compared between two trees? We resolved this issue by ‘ladderizing’ the trees: rotating branches around every node so that branches leading to the larger number of descendants (tips) were always on the same side. This procedure, conventionally used to produce a more aesthetically pleasing visualization of a phylogeny, greatly increases the chance of finding matching subset trees between two phylogenies. Furthermore, we rotated branches around all ‘cherries’ (nodes that are the direct ancestor of two tips) such that the longer branch was always on the same side. Based on simulations (see below), we determined that this combination of ladderization and rotating cherries significantly improved our ability to correctly classify simulated trees (Table S2), indicating that these steps resolved different trees into shapes which could be compared in a consistent and meaningful way.

Following Moschitti’s notation [16], we defined a phylogenetic tree shape kernel by:
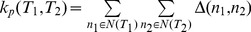
(1)where 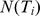 is the set of all nodes from the 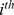 tree 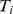, and 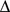 is defined as follows:

- If 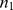 and 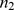 are leaf nodes, 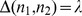, where 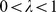 is a constant decay factor.
- If the numbers of children descending from 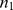 and 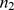 are the same (always two for bifurcating trees) and, of these, the numbers of leaf nodes are also the same, then:

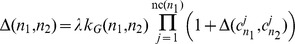
where 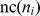 is the number of children descending from node 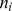, 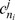 is the 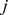-th child of node 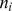, and 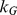 is a Gaussian radial basis function on the vectors of branch lengths descending from nodes 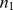 and 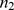, denoted as 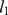 and 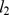 respectively:



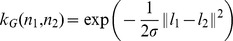
where 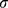 is the variance parameter that determines how strongly subset trees are penalized for discordant branch lengths.

- Otherwise, 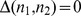.

Note that matching subset trees were weighted in proportion to their size (number of nodes). Because a tree will match its own shape exactly, 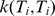 can take overwhelmingly large values. This ‘large diagonal’ effect [27] can severely curtail the effectiveness of subsequent analyses of the kernel matrix (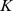), which is a symmetric matrix that is obtained by applying the kernel function to all pairs of 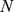 trees, 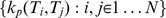. Therefore, the constant decay factor 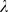 served to penalize larger subset trees because it declined exponentially with tree size [15]. The Gaussian radial basis function 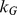 further penalized subset trees by the discordance in branch lengths for pairs of branches originating from the matching nodes (Figure 2). Although this incorporation of branch lengths is a significant departure from the original tree kernel for natural language processing, we prove that 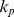 is a valid positive semi-definite kernel function (Text S1).

Phylogenies were imported from the Newick tree strings produced by RAxML into Python using the Phylo module from the Biopython package [28], [29]. Branch lengths in each phylogeny were normalized by the mean branch length to facilitate comparisons between viruses with different overall rates of evolution. A kernel matrix was computed from this collection of phylogenies using a custom Python module, which we have made publicly available on our webserver (http://bioinfo.cfenet.ubc.ca/pub/phylokernel). Although the kernel function can be computed quickly [16], we needed to compute the kernel matrix for 4153 phylogenies (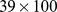 replicates + 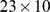 samples by collection year + 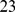) with over 8 million pairwise comparisons. To speed up this calculation, we performed parallel computation on a Linux cluster with a message-passing interface (MPI) environment [30]. The matrix was confirmed to be positive semi-definite by Cholesky decomposition. Following Collins and Duffy [15], all entries in the matrix were normalized by the formula
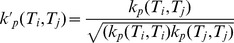
so that the resulting kernel matrix was less sensitive to differences in the sizes of the respective trees. Values of 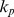 for replicate sets of phylogenies generated from random samples were averaged for every pairwise comparison of viruses or virus clades, and the resulting group mean distance matrix was renormalized. Kernel principal components analysis and support vector machine classification were performed on this normalized kernel matrix using the *R* package *kernlab* [31].

### Simulating Phylogenies

We used the R package *diversitree* [32] to simulate the growth of phylogenies under different evolutionary scenarios of branching or ‘speciation’ rates. Trees were generated under a Quantitative State Speciation and Extinction (QuaSSE) model [33] whereby speciation rates were determined by a continuous trait whose evolution was simulated by a Brownian motion (with zero drift and variance per unit time 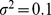) under two different mutation rates (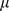 = 0.1 and 0.01). This continuous trait was mapped to a speciation rate by a sigmoidal function with a zero midpoint, exponential decay rate of 0.25, and minimum and maximum values of 0.05 and 0.25, respectively. We performed two sets of 100 replicate simulations under the different mutation rate scenarios. The simulated data were converted into Newick tree strings using a Python script derived from the Biopython Phylo module [29]. These strings are publicly available at http://bioinfo.cfenet.ubc.ca/pub/phylokernel. We calculated nine different tree balance statistics – Colless’ index, Sackin’s index, mean path length, variation in path length, Shao and Sokal’s 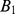 and 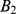, and the sum, total mean, and mean of the 10 earliest nodes of Fusco and Cronk’s imbalance statistic [34] – for all trees using functions written in Python. A kernel matrix was computed for all trees using the methods described above. We trained a 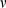-support vector machine classifier [35] using the R package *kernlab* on a random subset of half of the phylogenies, and then measured the sensitivity and specificity of classification by mutation rate on the remaining half. Likewise, the sensitivity and specificity of classifying trees by tree balance statistics was evaluated at varying cutoffs using the R package *ROCR* [36].

## Results

### Performance on Simulated Trees

We simulated two sets of 100 replicate phylogenies under different mutation rates (200 trees total) that influenced the branching rates in the phylogeny in an autocorrelated fashion [32]. In other words, the branching rate was allowed to evolve as each phylogeny grew from a common ancestor to 100 descendants. These simulations provide a reasonable approximation of the kind of evolutionary scenarios between which tree balance statistics were meant to differentiate [12], since preferential branching along fewer lineages will tend to produce an imbalanced tree. For all simulated phylogenies, we computed nine different balance statistics (the eight statistics assessed in [12] and Sackin’s index). In addition, we computed the kernel matrix for all simulated phylogenies using our phylogenetic tree shape kernel. Note that the kernel method does not function as a summary statistic; it cannot assign a value to a given tree in the absence of any other information. Consequently, we compared the kernel method to balance statistics by evaluating their performance at correctly classifying trees that had been generated under different models of speciation, which is analogous to the transmission of an infectious disease between hosts.

**Figure 3:**
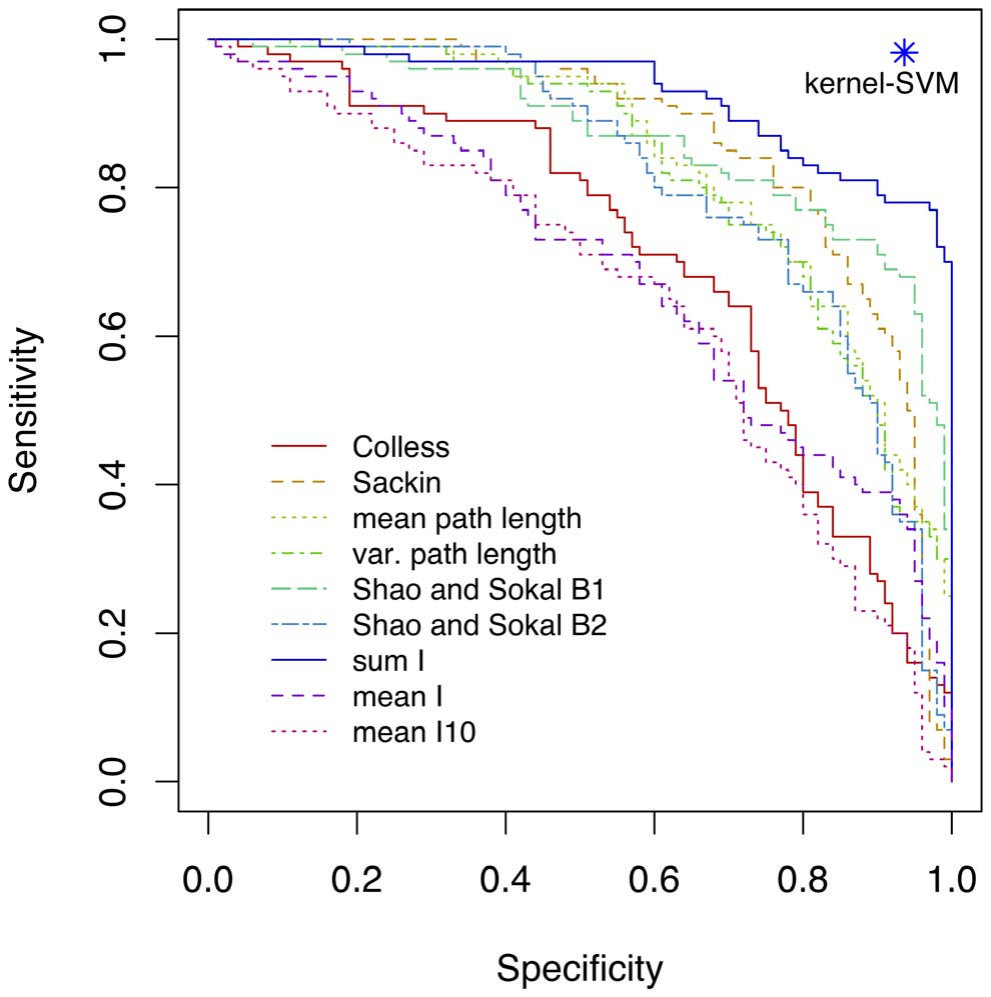
Classification of simulated phylogenies using nine balance statistics and the kernel function. We simulated the growth of two sets of 100 phylogenies relating 100 taxa under different scenarios in which rates of speciation (branching) evolved at different rates. Greater variation in speciation rates tended to produce more imbalanced trees. Nine different balance statistics, including eight from [12], were computed for all phylogenies: Colless’ index, Sackin’s index, the mean and variance in path lengths from tips to the root, Shao and Sokal’s 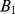 and 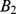 statistics, and the imbalance value (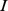) for the sum, total mean, and the mean of the earliest 10 internal nodes of the tree. This plot illustrates the trade-off between sensitivity and specificity of classifying phylogenies by applying a cutoff value each of these balance statistics. A single point (star) indicates the sensitivity and specificity attained by applying the phylogenetic kernel function (with 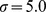 and 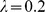) to train a support vector machine (SVM) on a random subset (50%) of the phylogenies, and classifying the remaining half.

The sensitivity and specificity of classifying the simulated phylogenies by mutation rates (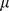 = 0.01 and 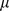 = 0.1) is summarized in Figure 3. Using our kernel method, we obtained a median sensitivity of 97.7% (interquartile range, IQR = 95.6%, 98.0%) and specificity of 90.8% (IQR = 89.1%, 92.8%) when averaged over 100 replicate training sets. These results were unambiguously superior to all nine balance statistics that were evaluated over the same simulated data. For instance, the sum of Fusco and Cronk’s imbalance statistic was the most effective among the balance statistics, but none could exceed a sensitivity of 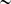
80% without a corresponding drop in specificity below 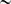
80%. Thus, the kernel method is capable of providing a substantial advantage for recognizing virus trees that have evolved under different evolutionary and epidemiological scenarios.

To determine the sensitivity of the kernel function to 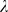 and 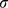, we evaluated our ability to classify simulated phylogenies under varying parameter settings. We found that both sensitivity and specificity were robust to different settings of 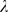 and 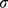 (Figure S1). For example, mean sensitivities varied by less than 1% (0.97 to 0.98) for values of 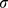 ranging from 1.0 to 20.0, given 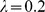; setting 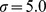 conferred a slight advantage over this range in the classification of simulated phylogenies. These tests indicated that results from applying our kernel function to RNA virus phylogenies would be robust to varying parameter settings. This conjecture was supported in a comparison of kernel matrix projections under different settings (Figure S2; see below).

### Tree Balance in RNA Viruses

We used maximum likelihood-based heuristics to reconstruct phylogenies from 62 sequence alignments representing 38 different human and zoonotic RNA viruses from 12 different taxonomic families (Table S1). Genetic sequences from viruses with highly divergent clades, such as the human immunodeficiency virus type 1 (HIV-1) subtypes or influenza A virus serotypes, were grouped accordingly into separate multiple sequence alignments to allow for clade-specific epidemiological or evolutionary dynamics. Replicate phylogenies were generated from random samples of 100 sequences (given adequate sample size) to control for the well-documented confounding effect of variation in sample size on conventional tree shape statistics [9].

**Figure 4:**
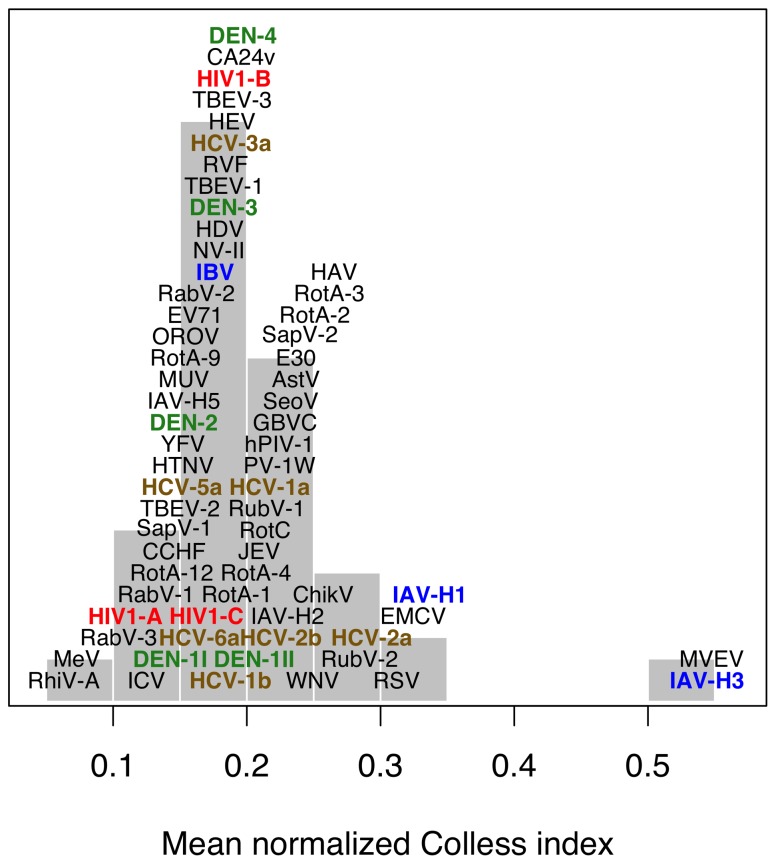
Distribution of mean normalized Colless’ indices. Each label represents the mean index of a virus or virus clade. The vertical axis is used to elucidate the clustering of points by forcing overlapping labels (phylogenies with similar indices) to ‘pile up’ like a histogram. A higher Colless’ index corresponds to a less ‘balanced’ tree in which branching events tend to occur along the same lineage. A conventional histogram is displayed in the background. Labels are defined as follows: AstV = *astrovirus*; CCHF = *Crimean-Congo hemorrhagic fever virus*; ChikV = *chikungunya virus*; CA24v = *coxsackievirus A24*; DEN = *dengue virus*; E30 = *echovirus 30*; EMCV = *encephalomyocarditis virus*; EV71 = *enterovirus 71*; GBVC = *GB virus C*; HTNV = *Hantaan virus*; H[A-E]V = *hepatitis [A-E] virus*; HIV = *human immunodeficiency virus type 1*; I[A-C]V = *influenza [A-C] virus*; JEV = *Japanese encephalitis virus*; MeV = *measles virus*; MuV = *mumps virus*; MVEV = *Murray valley encephalitis virus*; NV = *Norwalk virus*; OROV = *Oropouche virus*; hPIV-1 = *human parainfluenza virus*; PV = *poliovirus*; Rab = *rabies virus*; Rot = *human rotavirus*; RhiV = *human rhinovirus*; RSV = *human respiratory syncytical virus*; Rub = *rubella virus*; RVF = *Rift valley fever virus*; SapV = *sapovirus*; SeoV = *Seoul virus*; TBEV = *tick-borne encephalitis virus*; WNV = *West Nile virus*; YFV = *yellow fever virus*.

For each alignment, we calculated the means across replicates of two tree balance statistics (Colless’ index and Sackin’s index), which quantify the asymmetry in rates of branching among ancestral lineages in each tree. Specifically, Sackin’s index [8] counts the number of ancestral nodes separating each tip to the root, while Colless’ index [37] evaluates the difference in the number of tips that descend from the left and right branches of each bifurcating ancestral node. The distribution of mean Colless’ indices is displayed in Figure 4. As expected, influenza A virus (IAV) serotype H3 assumed one of the highest mean Colless’ indices (0.544) indicating that the replicate trees were severely imbalanced; only Murray valley encephalitis virus (MVEV) presented a higher index (0.548). Only 39 published MVEV envelope sequences were available after culling 7 identical sequences; however, Colless’ index is expected to be biased downwards with decreasing sample size, suggesting that the mean index for 100-sequence MVEV trees could have been even greater still. MVEV is endemic to Australia and Papua New Guinea and comprised almost entirely of mosquito isolates sampled over six decades (1956–2008). The IAV H3 sequences were strictly human isolates from around the globe but also sampled over a period of several decades (1968–2012).

This raises a concern of whether tree balance is an artefact of biased sampling of virus isolates over time, such that the overrepresentation of isolates from a narrow range of years might tend to yield balanced trees. To address this, we calculated the mean Colless’ indices for phylogenies generated from samples that were uniform with respect to sample collection years for alignments in which an adequate number of sequences were annotated with collection dates (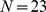). Using Lin’s concordance correlation coefficient (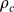) [38] to quantify the agreement between indices, where 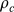 indicates perfect agreement and 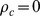 indicates no agreement, we found that the mean Colless’ indices for phylogenies whose tips were uniformly distributed over years were highly concordant with indices from random samples (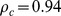, 95% C.I.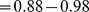). In other words, constraining the phylogenies of other viruses such as HIV to have evenly distributed tips over time did not make their Colless’ indices more IAV-like.

In sum, the distribution of mean Colless’ indices was highly peaked in the interval between 0.15 to 0.2, such that this summary statistic did not discriminate substantially among the phylogenies of most RNA viruses in our study (Figure 4). For example, the third-highest mean index (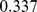) derived from IAV serotype H1 phylogenies was similar to the index of a hepatitis C virus (HCV) genotype 2a phylogeny (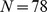), which would not seem to be consistent with the observation that phylogenies from HCV and IAV have conspicuously different shapes [2]. Specifically, we found that 6 out of 100 replicate samples of IAV-H1 sequences led to Colless’ indices that were less than the value observed for HCV-2a. Similarly, the relatively low Colless’ indices of phylogenies of influenza B virus (IBV, 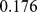), which together with IAV serotypes H1 and H3 is responsible for the majority of seasonal influenza outbreaks, implies that the imbalance of IBV-derived trees is no different from the bulk of animal RNA viruses (Figure 4). Furthermore, the mean Colless’ index did not differentiate the phylogenies of HIV and HCV from other RNA viruses. We obtained the same qualitative results from an evaluation of mean Sackin’s indices (Figure S3), which were highly correlated with the respective mean Colless’ indices (Pearson’s 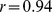, 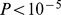). There is much more to the conspicuously star-like shape of HIV and HCV phylogenies that remains unquantifiable by balance statistics. Consequently, relying too heavily on tree balance statistics potentially limits our ability to measure the diversity of human and zoonotic RNA virus phylogenies, and to link recurring shapes to epidemiological or evolutionary processes.

### Kernel Analysis of RNA Virus Trees

Here, we develop and apply a new approach to comparing RNA virus tree shapes by using a method from machine learning known as the ‘kernel trick’ [10]. Rather than attempting to reduce a complex tree shape down to one or more summary statistics, like the tree balance indices examined above, we defined a kernel function (Figure 2) that efficiently computes inner products between phylogenies with respect to their tree shapes. By this approach, a tree’s shape is quantified only by its comparison to other trees. We have shown above that this method can be highly effective at discriminating between the shapes of phylogenies that were simulated under different evolutionary scenarios. We applied this phylogenetic tree kernel to our collection of RNA virus phylogenies to generate a kernel matrix that is akin to a pairwise similarity matrix. Entries in the kernel matrix were averaged across replicate phylogenies from the same alignments, and renormalized to yield a smaller kernel matrix with rows or columns corresponding to different viruses or virus clades and sampling schemes (completely at random or uniformly with respect to collection year). According to the eigenvalues of this projection, about 90% of the variance was explained by the first 2 principal components alone, with about 70% of the variance explained by the first component; these percentages varied slightly with different parameter settings of the kernel function (Figure S2). In all cases, the first component distinctly separated the phylogenies derived from HIV and HCV sequences from the rest of the RNA viruses. However, none of the remaining principal components were as readily interpretable. Furthermore, neither Sackin’s nor Colless’ indices were significantly correlated with any of the principal components based on a simple Pearson correlation test after adjusting for multiple comparisons. Additionally, we trained a support vector regression model using the kernel matrix and Colless’ indices for a random subset of the data. The mean concordance correlation coefficient [39] between the observed Colless’ indices and those predicted by this model for the remaining phylogenies was only 0.12 (interquartile range 0.07, 0.18), which is considered poor. These results, which were robust to varying 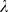 and 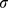, suggested that the kernel was manifesting aspects of phylogenetic tree shapes other than those captured by tree balance statistics.

**Figure 5:**
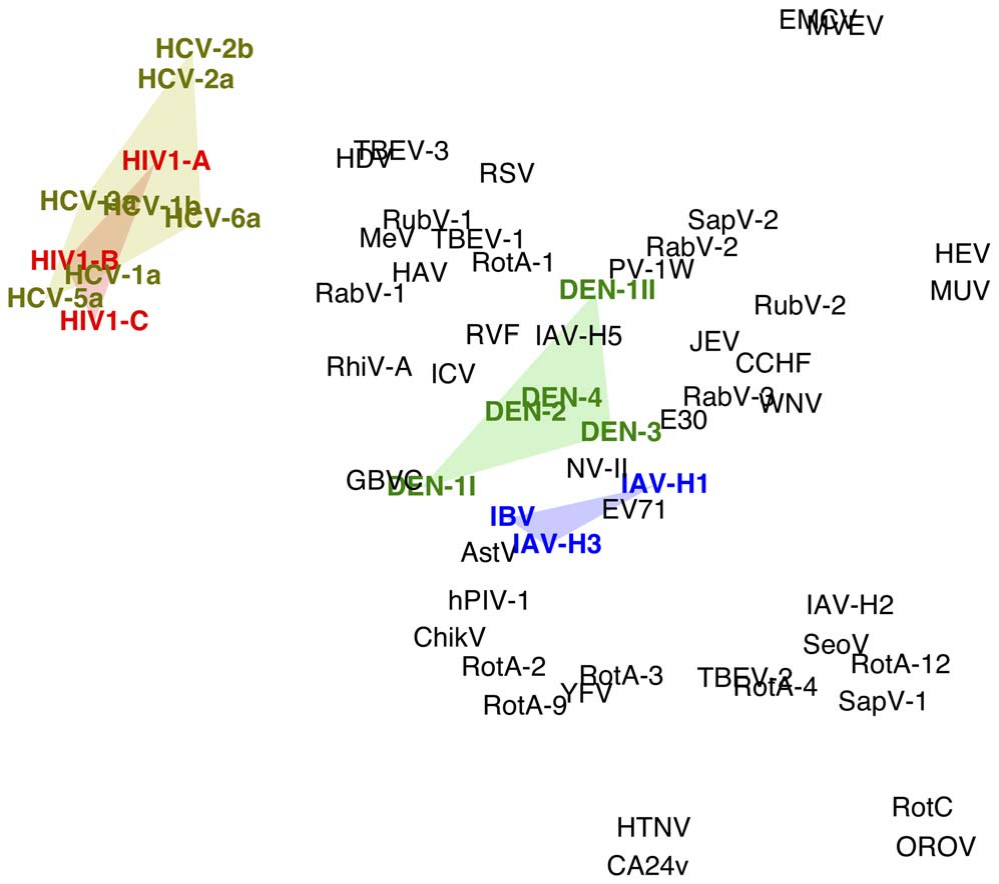
A visualization of RNA virus phylogenies in the tree shape kernel space (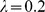, 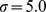) using t-distributed stochastic neighbor embedding (t-SNE). The t-SNE algorithm attempts to find the optimal map of high-dimensional data into a low-dimensional space while preserving the distances among points as much as possible. Thus, the distance between pair of viruses or virus clades (labelled by the same abbreviations as Figure 4) is approximately proportional to their mean kernel distance. Groups of virus clades of particular interest are highlighted with the corresponding colours: HIV, red; HCV, yellow; Dengue (DEN), green; IAV-H3, IAV-H1, and IBV (blue).

A scatterplot of the largest 2 principal components from the preceding analysis described a single arc in which HIV and HCV phylogenies comprised a distinct cluster from the other RNA viruses (Figure S2). However, the majority of the other viruses were agglomerated at the base of the arc, making it difficult to discern patterns from this visualization. This is a known issue in PCA in which the largest components are assumed to represent the important structure in the data whereas smaller components represent noise. To provide a clearer visualization, we generated another scatterplot (Figure 5) using a t-distributed stochastic neighbor embedding algorithm (t-SNE) which attempts to preserve both global and local structure in a low-dimensional visualization of high-dimensional data [40]. Again, HIV and HCV comprised a distinct cluster in this visualization. Phylogenies derived from these viruses tend to feature long terminal branches and relatively short internal branches (Figure 1), which has been attributed to the exponential spread of these viruses and their propensity to establish persistent infections. This outcome was robust to partitioning sequences by clade; for example, a phylogeny comprised of all three HIV subtypes in our study mapped adjacent to the HIV subtype-specific phylogenies in both types of projections (data not shown).

GB virus C (GBVC), which is not known to be associated with any human disease, was consistently located within the cluster of HIV and HCV phylogenies in the kernel PCA projections. (Due to the stochastic nature of the 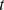-SNE algorithm, GBVC was not always embedded in the vicinity of HIV and HCV.) A close relative of HCV, GBVC can establish persistent infections in its human hosts and may inhibit HIV replication when an individual has become co-infected by both viruses [41]. Thus, the ability to establish persistent infections appears to be a common characteristic of viruses whose phylogenies map to this region of tree shape space. To test this hypothesis, we trained a support vector machine (SVM) classifier to differentiate between viruses having the ability to establish persistent chronic infections (HIV, HCV, and GBVC) and those without. We randomly selected half of the members from each category and used the corresponding rows and columns in the kernel matrix as training data. Validating the classifier on the remaining half of the kernel matrix, we consistently obtained a sensitivity and specificity of 1.0 irrespective of varying the kernel parameters 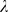 and 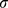, indicating that viruses that can establish persistent chronic infections were unambiguously separated from the other RNA viruses in the space defined by the tree kernel.

The influenza viruses predominantly responsible for the seasonal epidemics around the world (IAV-H1, IAV-H3, and IBV) were located close together in both projections of the kernel matrix. In contrast, the Sackin’s and Colless’ indices for IBV were substantially lower than those of IAV-H1 and IAV-H3. This suggests that the kernel was more successful in identifying commonalities in the shapes of the phylogenies of these influenza viruses. On the other hand, these influenza viruses were located in the midst of many other RNA viruses in both projections, despite the strikingly pectinate shape of IAV-H3 (Figure 1). Phylogenies from the other influenza viruses in this study (IAV-H2, IAV-H5, and influenza C virus) mapped further away from the seasonal epidemic influenza viruses. These other phylogenies tended to have longer internal branches (relative to terminal branches) than the phylogenies of IAV-H1, IAV-H3, and IBV. Specifically, the ratio of the median branch lengths of internal and terminal branches, respectively, was significantly greater for the other influenza viruses than the seasonal epidemic influenza viruses (Wilcoxon rank sum test 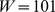, 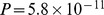). A possible explanation for this pattern is that the H2 and H5 subtypes are infrequently re-introduced into the human population from under-sampled avian host populations; consequently, the evolving diversity of these viruses would be represented by only a small number of lineages in phylogenies.

Our survey of animal RNA viruses included several examples of zoonotic viruses. One might anticipate that these viruses could map to one or more distinct regions in projections of the kernel matrix. We observed that mosquito-transmitted zoonotic viruses such as the highly divergent dengue serotypes (DEN-1 to -4), the arboviral encephalitides (Japanese encephalitis virus and West Nile virus), and yellow fever virus tended to map to a similar region in projections of the kernel matrix, although this region was not exclusive of other viruses for which humans are the only known natural hosts, such as rubella virus and poliovirus (Figure 5). Another interesting feature of this projection was that phylogenies representing clades of the zoonotic rabies virus (RabV-1 to -3) mapped to a similar region as the mosquito-transmitted zoonotic viruses. The majority of the rabies virus sequences were isolated from non-human hosts predominated by canine (numbered here as clades 1 and 3) and bat species (clade 2), with about 2%–9% sequences isolated from human hosts (Table S1).

Finally, we observed that phylogenies from certain viruses shared a distinctive rapid proliferation of short terminal branches within one or more specific clades which may be a signature of sporadic outbreaks, as illustrated by the coxsackievirus A24 variant phylogeny (CA24v) depicted in Figure 1. For example, the majority of CA24v sequences within the highly unresolved clade of its phylogenies were sampled during outbreaks of acute hemorrhagic conjunctivitis in Brazil and China. The proximity of the zoonotic viruses Hantaan virus (HTNV) and Murray Valley encephalitis virus (MVEV) to CA24v in projections of the kernel matrices (Figure S2) suggested that the shapes of the respective phylogenies may have also been shaped by sporadic outbreaks.

To assess the potential bias of uneven sampling over time, we incorporated replicate phylogenies into the kernel matrix that were generated from samples of virus sequences drawn at random with respect to collection year. We found that the mean distances between phylogenies sampled completely at random and phylogenies sampled uniformly by collection year from the same virus or virus clade were significantly shorter (larger kernel scores) than the mean distances between phylogenies from different viruses or virus clades (Wilcoxon rank sum test, 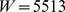, 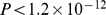). Specifically, the mean normalized kernel score between phylogenies of the same virus obtained by different sampling schemes was 0.98, whereas the mean among phylogenies from different viruses was 0.84, where a normalized score of 1.0 indicates an exact match with respect to tree shape. On visually examining a PCA projection of this kernel matrix, we found that sampling uniformly by collection year tended to shift phylogenies along the same trajectory in the space of tree shapes (Figure S4). With the exception of HCV genotype 6a (for which about 50% of isolates were obtained in 2006 alone), the cluster of HIV and HCV viruses remained distinct from other RNA viruses including the influenza viruses.

## Discussion

In this study, we have proposed a new framework for comparing phylogenetic tree shapes by extending a parse tree kernel from computational linguistics [16]. Our kernel function compares all subset trees shared by two phylogenies and weights this comparison by the concordance of branch lengths that comprise the respective subset trees (Figure 2). It therefore bears some similarity to the weighted Robinson-Foulds metric that is based on comparing the lengths of branches that descend from each ancestral node [14]. However, the Robinson-Foulds metric is restricted to comparing alternative trees relating the same taxa; in other words, the terminal branches must have the same labels. This requirement makes it impossible to use the Robinson-Foulds metric to compare phylogenies from different viruses. In contrast, our kernel is designed to compare trees from completely different sets of taxa because it discriminates among subset trees primarily by the number of terminal branches that descend from each ancestral node, regardless of what taxa occupy the tips of those branches. Moreover, if two phylogenies had a subset tree in common, that subset tree would have to occur in the same location of the trees to be counted towards the Robinson-Foulds metric. In contrast, since the tree shape kernel iterates over all internal nodes in both phylogenies, subset trees in different locations can contribute towards the kernel score. This is an important distinction because we are primarily interested in recurring motifs in tree shape, irrespective of where they appear in either tree, because this commonality may represent the imprint of a common epidemiological or evolutionary process acting on both viruses.

Using conventional tree balance statistics, we found limited variation among phylogenies from different RNA viruses, with most viruses falling into a narrow range of Colless’ or Sackin’s indices after controlling for sample size variation. In contrast, our kernel function was able to separate the distinctively star-like phylogenies of HIV and hepatitis C virus. However, it was unable to distinguish the conspicuously pectinate shapes of IAV-H1 and -H3 phylogenies to the same extent as the tree balance statistics. This is most likely due to our use of a decay factor 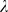 in computing the kernel function to penalize larger subset tree matches. Unless we penalize subset trees by 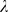, large phylogenies would result in enormous values along the diagonal of the kernel matrix because a phylogeny will always match itself exactly, washing out any patterns from comparisons between different phylogenies. In other words, our use of a decay factor places greater emphasis on local than global similarities between the shapes of two phylogenies. Using a decay factor evidently prevents the full extent of the imbalance in the IAV-H1 or -H3 phylogenies from being expressed in the kernel matrix, since the longer subset trees that would represent this large-scale pattern are penalized more heavily.

A common concern directed at studies of phylogenetic tree shapes is that variation in tree shapes may be an artefact of differences in sampling. For example, influenza A virus sequences in our data were sampled over a wide range of years from 1925 to 2012, with 72% of sequences sampled after 2001. It is possible that differences among viruses in the distributions of sample dates may introduce a bias to tree balance statistics or the kernel method. However, when we constrained samples across viruses to be sampled uniformly with respect to collection years, we found no significant effect on the distributions of RNA viruses for either tree balance or kernel-based methods. Analyses of tree shapes can be robust to temporal sampling biases because the internal structures of the phylogenies are often comprised of ancestors that are substantially further back in time. For example, there was very little clustering of HIV-1 subtype B sequences in the phylogeny with respect to collection year, implying that a phylogeny reconstructed a tree from sequences collected in a specific year will retain most of its deeper structure.

The tree shape kernel presented here potentially represents an important new exploratory tool for studying RNA virus phylogenies. By extracting recurring local motifs in the internal structures of different phylogenies, it can complement the established tree balance statistics to provide a more complete description of the diversity of tree shapes among RNA viruses [3]. Our kernel is not a parametric model in itself; unlike recent advances in viral phylodynamics [42], it does not attempt to directly estimate epidemiological model parameters from tree shapes. Unlike a tree balance statistic, the kernel function cannot assign a numerical value to a single phylogeny in the absence of any other data. Without summary statistics to compare to reported values in the literature, it is necessary to have at hand all the phylogenies that one wishes to compare in order to compute the entire kernel matrix. On the other hand, the use of a kernel method presents opportunities to utilize other techniques from machine learning such as support vector machines (SVMs) to extract and analyze patterns in phylogenetic tree shapes. For example, we simulated phylogenies under two different evolutionary scenarios that influenced the asymmetry in branching rates among lineages, and we found that an SVM assisted by our kernel function clearly outperformed nine different tree balance statistics at predicting which phylogeny was generated under which scenario (Figure 3). Furthermore, we trained another SVM classifier on a random subset of phylogenies labelled by whether the respective RNA viruses could establish persistent chronic infections. The trained model consistently predicted these labels for the remaining phylogenies with very high sensitivity and specificity (see Results section).

It is possible that a similar procedure could be used, for example, to determine the natural animal reservoir of an incompletely characterized zoonotic virus. This may not only require further development of kernel methods such as those presented here, but also greater coverage and more detailed annotation of published sequence data for RNA viruses that are under-represented in public databases. Additionally, the tree shape kernel may provide an effective distance measure for fitting complex phylodynamic models by approximate Bayesian computation [43], a rapidly emerging class of highly versatile and efficient computational methods in which a distance measure (such as the difference in summary statistics) is used to assess the congruence between model simulations and observed data.

## Supporting Information

Figure S1
**Sensitivities (solid lines) and specificities (dashed lines) of classifying simulated phylogenies where the kernel matrix was generated under different settings of** 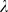 **and** 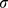 **.** Classification was performed using a kernel 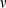-support vector machine implemented in the *R* package *kernlab* [31]. For a given kernel matrix, sensitivity and specificity were averaged over 1000 replicate cross-validations on random samples of trees stratified by evolutionary scenario. Note that the 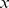-axis is 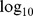-transformed and the 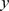-axis is scaled to the range of the observed values, which all exceeded the performance of classifications based on tree balance statistics. Varying the decay factor 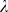 had little effect on either sensitivity or specificity; setting 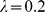 conferred a very slight advantage in sensitivity over 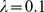 or 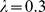. Both sensitivity and specificity were fairly robust to a wide range of values for the Gaussian radial basis function variance parameter 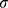. For example, mean sensitivities varied by less than 1% for values of 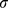 ranging from 1 to 20.(PDF)

Figure S2
**PCA projections for kernel matrices generated from the RNA virus phylogenies under different settings for kernel function parameters** 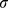 **and** 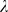 **.** Labels corresponding to virus species and clades are the same in Figure 4. Viruses of particular interest (such as HIV and the common pandemic influenza A viruses) are highlighted with coloured convex hulls as in Figure 5. The proportion of variance explained by the first two principal components (roughly 90% to 95%), as determined by eigenvalues, are reported as percentages in the axes labels.(PDF)

Figure S3 **Distribution of mean normalized Sackin’s indices.** Each label represents the mean index of a virus or virus clade. The vertical axis is used to elucidate the clustering of points by forcing overlapping labels (phylogenies with similar indices) to ‘pile up’ like a histogram. A higher Sackin index corresponds to a less ‘balanced’ tree in which branching events tend to occur along the same lineage. Label annotations are identical to Figure 4.
(PDF)

Figure S4
**Visualizing the effect of resampling virus sequences uniformly at random with respect to year of collection.** The kernel matrix calculated from the original sample phylogenies (black) and the additional phylogenies from uniform sampling by year (red) is projected onto the first two principal components. Arrows are drawn between phylogenies generated by different sampling schemes from the same set of virus sequences. Note that this PCA projection is slightly different from projection generated under the same settings (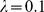, 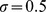) depicted in Figure S2 because it incorporates the uniform sample points.(PDF)

Table S1 **A list of all animal RNA viruses in this study, grouped by taxonomic family (bold face).** Abbrev. = abbreviation used in figures. *N* = number of sequences. %human = percentage of sequences isolated from human hosts. *L* = length of alignment in nucleotides. Outgroup accession = Genbank accession number of sequence used as outgroup. Reference = citation of article in peer-reviewed literature on which choice of outgroup is based; or a short note on the method used for outgroup selection.
(PDF)

Table S2
**Performance of kernel support vector machine classifier on 200 simulated phylogenies.** Trees were simulated with the R package *diversitree* by evolving a latent character state (with mutation rates 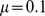 and 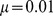) which controlled the rate of speciation (branching). All trees were modified by rotating branches around nodes according to some predefined scheme, resulting in different but evolutionarily invariant shapes. Kernel matrices were generated with the parameter settings 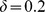 and 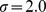. Sensitivity and specificity values were generated by R package *ROCR* and averaged across 1000 cross-validations (using a random subset of 100 trees to train the kernel classifier and validating on the remaining 100). Empirical 95% confidence intervals (C.I.) were derived from the 25th and 975th-ranked cross validations. Randomly rotating branches in the trees did not significantly affect our ability to classify them by evolutionary scenario (Student’s 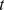-test, 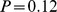). Ladderizing the trees, such that the most prolific branches were rotated to the same side, conferred a substantial and significant gain in sensitivity and specificity of classification relative to the unmodified trees (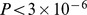). Rotating ‘cherries’ (pairs of tips that descend directly from their common ancestor) of a ladderized tree, so that the longest branch was always to the same side, conferred a slight but significant advantage in classification (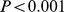). However, we found no advantage to rotating branches around internal nodes according to any of the schemes we evaluated. ‘Ties’ refer to nodes that are not cherries and have the same number of descendant tips to the left and right, making them ambiguous to ladderization. Rotating branches ‘by subtree’ indicates that the branch with the largest total branch length in the descendant subtree was rotated to the same side.(PDF)

Text S1
**Proof that** 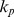 **is a positive semidefinite kernel.**
(PDF)
