# Supplementary figures and images for: Mapping the Shapes of Phylogenetic Trees from Human and Zoonotic RNA Viruses
Source: PLoS One. 2013 Nov 1;8(11):e78122. doi: 10.1371/journal.pone.0078122 (PMC3815201; doi:10.1371/journal.pone.0078122)

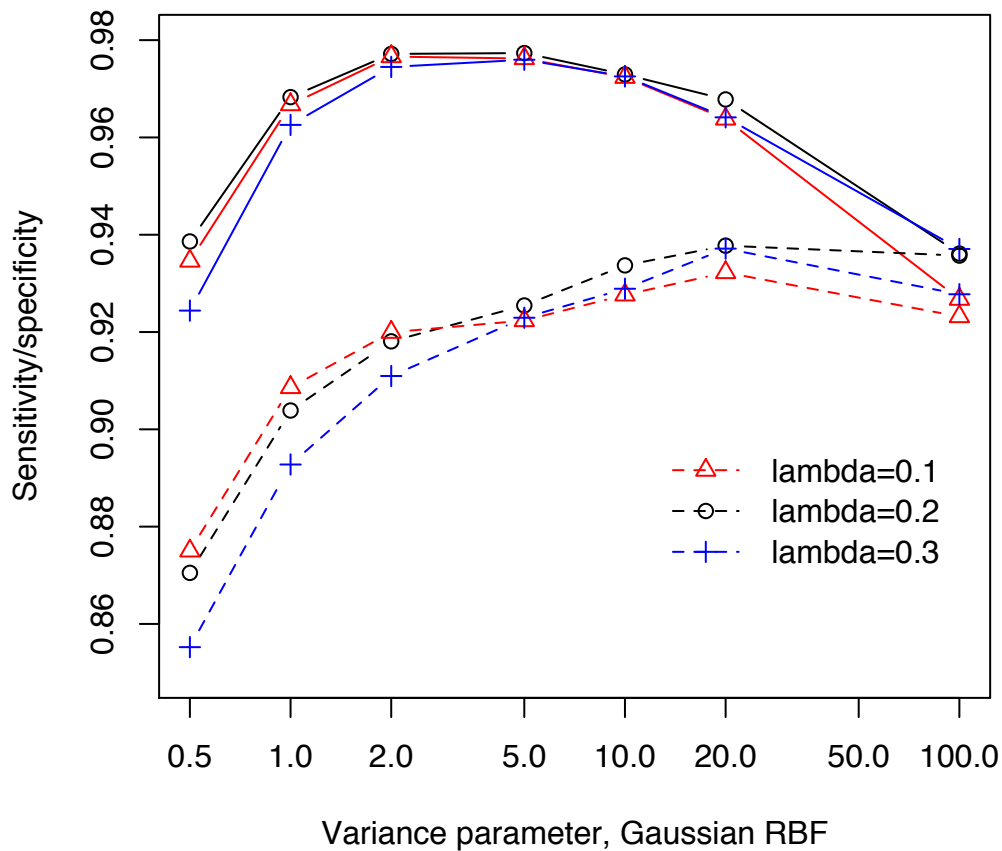

Supplement: Figure S1 — Sensitivities (solid lines) and specificities (dashed lines) of classifying simulated phylogenies where the kernel matrix was generated under different settings of and . Classification was performed using a kernel -support vector machine implemented in the R package kernlab [31]. For a given kernel matrix, sensitivity and specificity were averaged over 1000 replicate cross-validations on random samples of trees stratified by evolutionary scenario. Note that the -axis is -transformed and the -axis is scaled to the range of the observed values, which all exceeded the performance of classifications based on tree balance statistics. Varying the decay factor had little effect on either sensitivity or specificity; setting conferred a very slight advantage in sensitivity over or . Both sensitivity and specificity were fairly robust to a wide range of values for the Gaussian radial basis function variance parameter . For example, mean sensitivities varied by less than 1% for values of ranging from 1 to 20. (PDF) [file pone.0078122.s001.pdf]

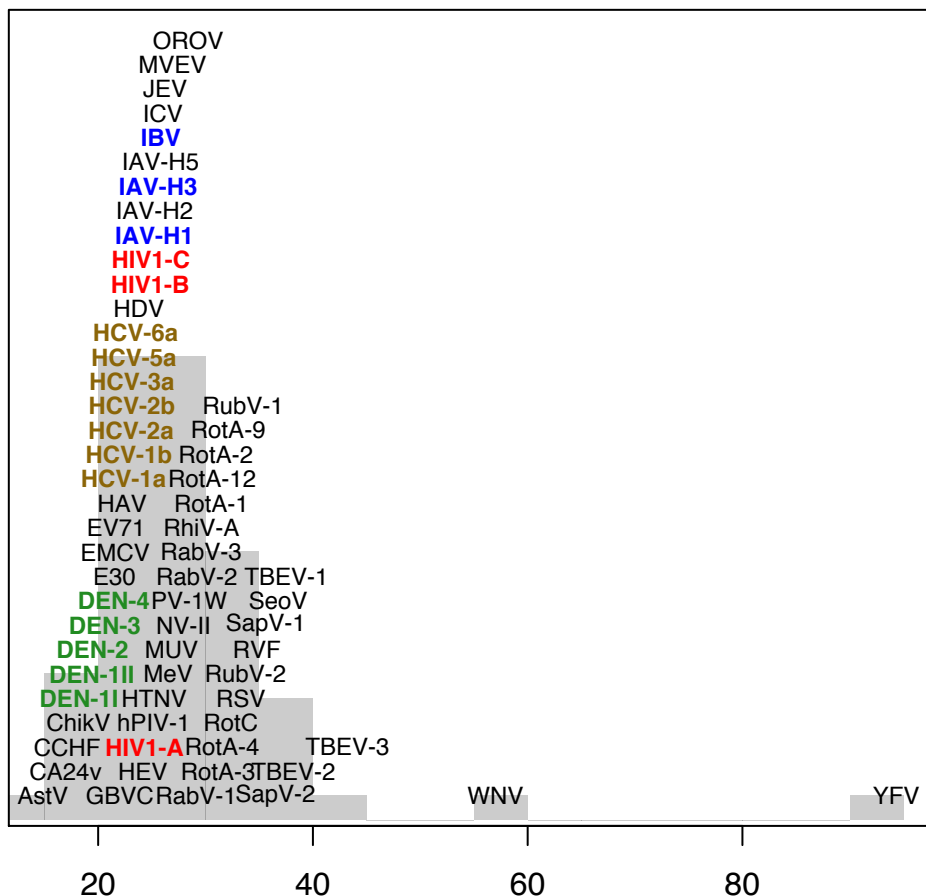

Mean normalized Sackin index

Supplement: Figure S3 — Distribution of mean normalized Sackin’s indices. Each label represents the mean index of a virus or virus clade. The vertical axis is used to elucidate the clustering of points by forcing overlapping labels (phylogenies with similar indices) to ‘pile up’ like a histogram. A higher Sackin index corresponds to a less ‘balanced’ tree in which branching events tend to occur along the same lineage. Label annotations are identical to Figure 4. (PDF) [file pone.0078122.s003.pdf]

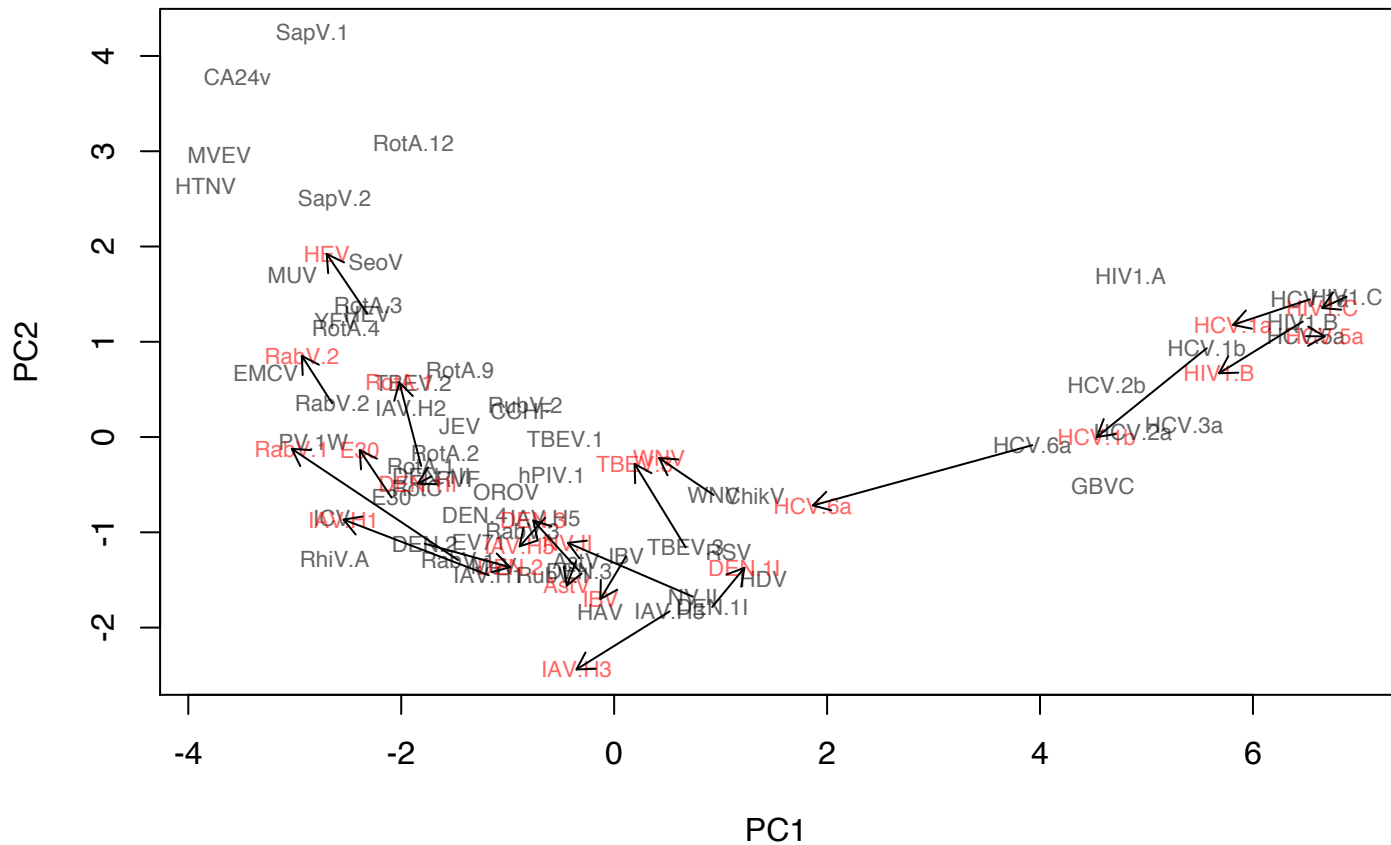

Supplement: Figure S4 — Visualizing the effect of resampling virus sequences uniformly at random with respect to year of collection. The kernel matrix calculated from the original sample phylogenies (black) and the additional phylogenies from uniform sampling by year (red) is projected onto the first two principal components. Arrows are drawn between phylogenies generated by different sampling schemes from the same set of virus sequences. Note that this PCA projection is slightly different from projection generated under the same settings (, ) depicted in Figure S2 because it incorporates the uniform sample points. (PDF) [file pone.0078122.s004.pdf]
