# Supplementary material for: Mapping the Shapes of Phylogenetic Trees from Human and Zoonotic RNA Viruses
Source: PLoS One. 2013 Nov 1;8(11):e78122. doi: 10.1371/journal.pone.0078122 (PMC3815201; doi:10.1371/journal.pone.0078122)
Supplement: Table S2 — Performance of kernel support vector machine classifier on 200 simulated phylogenies. Trees were simulated with the R package diversitree by evolving a latent character state (with mutation rates and ) which controlled the rate of speciation (branching). All trees were modified by rotating branches around nodes according to some predefined scheme, resulting in different but evolutionarily invariant shapes. Kernel matrices were generated with the parameter settings and . Sensitivity and specificity values were generated by R package ROCR and averaged across 1000 cross-validations (using a random subset of 100 trees to train the kernel classifier and validating on the remaining 100). Empirical 95% confidence intervals (C.I.) were derived from the 25th and 975th-ranked cross validations. Randomly rotating branches in the trees did not significantly affect our ability to classify them by evolutionary scenario (Student’s -test, ). Ladderizing the trees, such that the most prolific branches were rotated to the same side, conferred a substantial and significant gain in sensitivity and specificity of classification relative to the unmodified trees (). Rotating ‘cherries’ (pairs of tips that descend directly from their common ancestor) of a ladderized tree, so that the longest branch was always to the same side, conferred a slight but significant advantage in classification (). However, we found no advantage to rotating branches around internal nodes according to any of the schemes we evaluated. ‘Ties’ refer to nodes that are not cherries and have the same number of descendant tips to the left and right, making them ambiguous to ladderization. Rotating branches ‘by subtree’ indicates that the branch with the largest total branch length in the descendant subtree was rotated to the same side. (PDF) [file pone.0078122.s006.pdf]

Supporting Table S2

Setting	Sensitivity (95% C.I.)	Specificity (95% C.I.)
Unmodified	0.879 (0.767, 0.977)	0.854 (0.772, 0.933)
Randomized	0.869 (0.766, 0.957)	0.851 (0.759, 0.940)
Ladderized	0.976 (0.918, 1.000)	0.919 (0.852, 0.980)
Rotating cherries	0.981 (0.938, 1.000)	0.925 (0.860, 0.980)
Rotating nodes with ties	0.979 (0.935, 1.000)	0.922 (0.857, 0.980)
Rotating all nodes	0.967 (0.889, 1.000)	0.909 (0.842, 0.979)
Rotating nodes with ties by subtree	0.976 (0.920, 1.000)	0.918 (0.847, 0.980)
Rotating all nodes by subtree	0.878 (0.764, 0.974)	0.856 (0.766, 0.936)
