# Supplementary material for: Mapping the Shapes of Phylogenetic Trees from Human and Zoonotic RNA Viruses
Source: PLoS One. 2013 Nov 1;8(11):e78122. doi: 10.1371/journal.pone.0078122 (PMC3815201; doi:10.1371/journal.pone.0078122)
Supplement: Text S1 — Proof that is a positive semidefinite kernel. (PDF) [file pone.0078122.s007.pdf]

Text S1: Proof that k_p is a positive semidefinite kernel

We must show that k_p is positive semidefinite in order to rigorously use it in kernel-based machine learning algorithms. First, we need some notation. Let $k_G^n : \mathbb{R}^n \times \mathbb{R}^n \longrightarrow \mathbb{R}$ denote the Gaussian radial basis function (RBF) kernel on $\mathbb{R}^n$: if $x, x' \in \mathbb{R}^n$,

$$k_G^n(x, x') = \exp\left(-\frac{d(x, x')^2}{2\sigma^2}\right)$$

where d is the usual Euclidean distance in $\mathbb{R}^n$ and σ^2 is the variance parameter. To clean up our notation we will omit the superscript n from k_G^n when it is clear from the parameters what dimension we are working in. Recall that our kernel $k_p(T_1, T_2)$ is defined as $\sum_{n_1 \in N(T_1)} \sum_{n_2 \in N(T_2)} \Delta(n_1, n_2)$. To get a handle on the intuition behind this kernel, first observe that $\Delta(n_1, n_2)$ returns the sum of the Gaussian RBF kernel distances between the branch lengths of every pair of common subset trees rooted at n_1 and n_2. This uses the fact that

$$\begin{aligned} &k_G^m((x_1, \ldots, x_m), (x_1', \ldots, x_m'))\, k_G^n((y_1, \ldots, y_n), (y_1', \ldots, y_n')) \\ &= k_G^{m+n}((x_1, \ldots, x_m, y_1, \ldots, y_n), (x_1', \ldots, x_m', y_1', \ldots, y_n')). \end{aligned}$$

Thus k_p returns the sum of the Gaussian RBF kernel distances between the lengths of every subset tree common to T_1 and T_2.

In order to show that k_p is positive semi-definite, it suffices to show that $k_p(T_1, T_2)$ may be written as an inner product in some linear space which we map the trees to. Similar to the subtree- and subset tree-kernels used in natural language processing [1], we use a mapping that maps a tree to an element that summarizes aspects of the tree. First, a technical detail: as with any positive semidefinite kernel, the Gaussian RBF kernel in n dimensions may also be interpreted as an inner product on a vector space $\mathcal{G}_n$ after elements of $\mathbb{R}^n$ have been mapped to $\mathcal{G}_n$ via some mapping which we will call ψ_{RBF} (again, we omit mentioning n as it will be clear from the parameters what dimension we mean). This space is comprised of functions satisfying some constraint on their Fourier transforms [2]. We will use the spaces $\mathcal{G}_n$ frequently in the construction of our summary space $\mathcal{A}$.

Let a *feature* refer to a certain tree shape, ignoring branch lengths for the time being. A tree is said to have a feature if this tree shape exists as a subset tree within the tree, regardless of the branch lengths of the feature. Let $\mathcal{F}$ be the set of all possible tree features. (For convenience, we can take this set to be finite since we will only ever deal with two finite trees at a time.) The summary space $\mathcal{A}$ is defined as

$$\mathcal{A} = \left\{\text{maps } (F, k) \mapsto \left(\mathcal{G}_{|F|}\right)^k, \text{ where } F \text{ is a feature and } k \in \mathbb{N}\right\};$$

in other words, maps from features to vectors of elements of $\mathcal{G}_{|F|}$. Let γ be our mapping from trees into $\mathcal{A}$; we define it as follows. If a feature F occurs within a tree T exactly k times, then $\gamma(T)(F, k)$ will be a k-vector of elements of $\mathcal{G}_{|F|}$. Each element of this vector corresponds to one occurrence of F: it represents its branch lengths under the mapping ψ_{RBF}. For any other $n \neq k$,

$\gamma(T)(F,n) = 0$, where we use 0 to denote the 0 element of $\mathcal{G}^k_{|F|}$. Similarly, for any feature F' that doesn't exist in the tree, $\gamma(T)(F',\cdot) \equiv 0$. Note that under our mapping, we will never get an element of $\mathcal{A}$ with more than one non-zero value in $(F,1),(F,2),\ldots$, since a tree cannot contain a feature exactly k times for more than one value of k. However, we need to define $\mathcal{A}$ this way in order for it to be an appropriate vector space.

It can be shown that $\mathcal{A}$ is a real vector space; it remains to define an inner product on $\mathcal{A}$. Let $\lambda, \mu \in \mathcal{A}$. We define

$$\lambda * \mu = \sum_{F\in\mathcal{F}} \sum_{k_1\in\mathbb{N}} \sum_{k_2\in\mathbb{N}} \sum_{i=1}^{k_1} \sum_{j=1}^{k_2} \langle \lambda(F,k_1)_i, \mu(F,k_2)_j \rangle_{\mathcal{G}_{|F|}},$$

where $\langle\cdot,\cdot\rangle_{\mathcal{G}_{|F|}}$ is the inner product in $\mathcal{G}_{|F|}$. The properties of an inner product follow from the fact that $\langle\cdot,\cdot\rangle_{\mathcal{G}_{|F|}}$ is an inner product.

This inner product is only intended to be meaningful when applied to elements in the image of γ, so we consider this case more closely. Recall that for a given tree T, we can never have $\gamma(T)(F,k)$ and $\gamma(T)(F,k')$ both non-zero when $k \neq k'$. Let T_1 and T_2 be trees, and say that the number of times a feature F occurs in T_i is $k_{i,F}$. In this case, the sum reduces to

$$\begin{aligned}
&\gamma(T_1) * \gamma(T_2) \\
&= \sum_{F\in\mathcal{F}} \sum_{i=1}^{k_{1,F}} \sum_{j=1}^{k_{2,F}} \langle \gamma(T_1)(F,k_{1,F})_i, \gamma(T_2)(F,k_{2,F})_j \rangle_{\mathcal{G}_{|F|}} \\
&= \sum_{F\in\mathcal{F}} \sum_{i=1}^{k_{1,F}} \sum_{j=1}^{k_{2,F}} k_G((i\text{th occurrence of } F \text{ in } T_1), (i\text{th occurrence of } F \text{ in } T_2)).
\end{aligned}$$

This sum includes a term for every pair of subset trees common to T_1 and T_2, and thus it equals $k_p(T_1,T_2)$ as defined by equation (1) in the main text. This shows that $k_p(T_1,T_2) = \gamma(T_1) * \gamma(T_2)$, and thus is positive semidefinite.

References

1. Collins M, Duffy N (2002) Convolution kernels for natural language. In: Dietterich TG, Becker S, Ghahramani Z, editors, Advances in Neural Information Processing Systems 14. Cambridge, MA: MIT Press.

2. Vert JP, Tsuda K, Schölkopf B (2004) Kernel methods in computational biology, MIT Press, chapter 2. pp. 35-70.
