# Supplementary material for: Mapping the Shapes of Phylogenetic Trees from Human and Zoonotic RNA Viruses
Source: PLoS One. 2013 Nov 1;8(11):e78122. doi: 10.1371/journal.pone.0078122 (PMC3815201; doi:10.1371/journal.pone.0078122)
Supplement: Figure S2 — PCA projections for kernel matrices generated from the RNA virus phylogenies under different settings for kernel function parameters and . Labels corresponding to virus species and clades are the same in Figure 4. Viruses of particular interest (such as HIV and the common pandemic influenza A viruses) are highlighted with coloured convex hulls as in Figure 5. The proportion of variance explained by the first two principal components (roughly 90% to 95%), as determined by eigenvalues, are reported as percentages in the axes labels. (PDF) [file pone.0078122.s002.pdf]

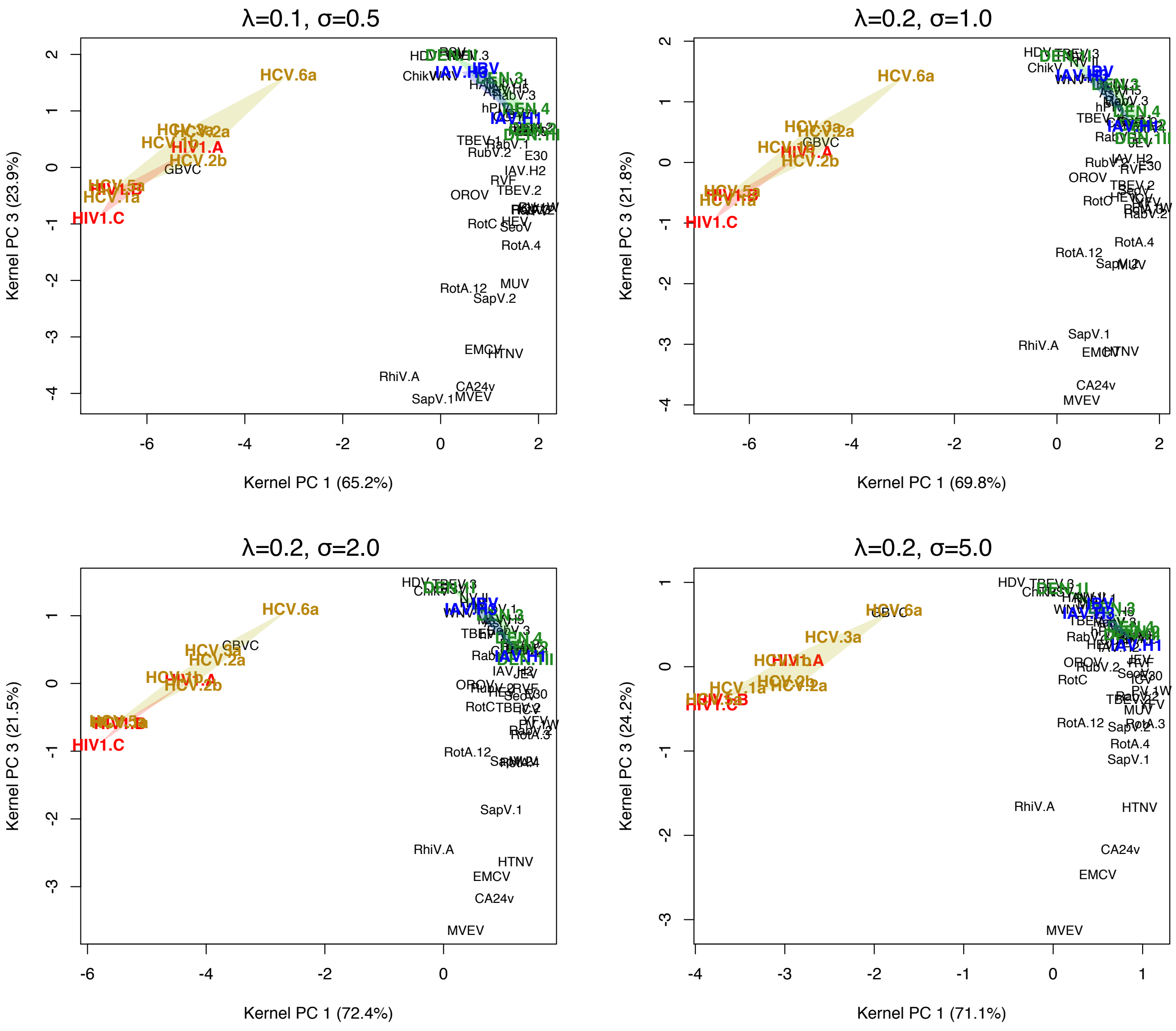
λ=0.1, σ=0.5
Kernel PC 3 (23.9%)
Kernel PC 1 (65.2%)
λ=0.2, σ=1.0
Kernel PC 3 (21.8%)
Kernel PC 1 (69.8%)
λ=0.2, σ=2.0
Kernel PC 3 (21.5%)
Kernel PC 1 (72.4%)
λ=0.2, σ=5.0
Kernel PC 3 (24.2%)
Kernel PC 1 (71.1%)
HCV.6a
HCV.2b
HCV.3a
HCV.2a
HCV.1a
HCV.5a
HIV1.A
HIV1.B
HIV1.C
GBVC
RhiV.A
SapV.1
SapV.2
MVEV
CA24v
EMCV
HTNV
MUV
RotA.12
RotA.4
RotA.3
RotC
OROV
RVF
IAV.H2
IAV.H1
IAV.H3
IBV
TBEV.2
TBEV.3
HDV
ChikV
WNV
hPIV
RabV.1
RabV.2
RabV.3
RubV.2
E30
SeoV
HEV
ICV
YFV
PV.1W
JEV
DEN.3
DEN.4
DEN.1II
