# Supplementary material for: Mapping the Shapes of Phylogenetic Trees from Human and Zoonotic RNA Viruses
Source: PLoS One. 2013 Nov 1;8(11):e78122. doi: 10.1371/journal.pone.0078122 (PMC3815201; doi:10.1371/journal.pone.0078122)
Supplement: Table S1 — A list of all animal RNA viruses in this study, grouped by taxonomic family (bold face). Abbrev. = abbreviation used in figures. N = number of sequences. %human = percentage of sequences isolated from human hosts. L = length of alignment in nucleotides. Outgroup accession = Genbank accession number of sequence used as outgroup. Reference = citation of article in peer-reviewed literature on which choice of outgroup is based; or a short note on the method used for outgroup selection. (PDF) [file pone.0078122.s005.pdf]

Virus	Subtype/ genotype/ serotype	Abbrev.	*N*	% human	Gene/product	*L*	Outgroup accession	Ref.
**Astroviridae**								
Astrovirus		AstV	350	100.00	capsid	348	AF056197	[1]
**Bunyaviridae**								
Hantaan virus		HTNV	109	7.06	glycoprotein G2	357	NC_005234	[2]
Oropouche virus		OROV	98	95.08	nucleocapsid (N)	693	AY383717	[3]
Rift valley fever virus		RVF	171	41.61	glycoprotein	718	DQ380191	[4]
Seoul virus		SeoV	234	6.33	nucleocapsid	415	JQ665905	[5]
**Calciviridae**								
Norwalk virus	II	NV-II	743	99.13	capsid protein (VP1)	1623	JX846924	[6]
Sapovirus	1	SapV-1	180	94.74	capsid protein	729	FJ844411	[7]
Sapovirus	2	SapV-2	55	95.24		729	AY646853	
**Flaviviridae**								
Dengue virus	1I	DEN-1I	1055	99.17	E protein (envelope)	1485	AB074760	[8]
Dengue virus	1II	DEN-1II	449	93.83		1485	AB074760	
Dengue virus	2	DEN-2	1022	95.08		1487	D00346	[9]
Dengue virus	3	DEN-3	1008	97.96		1500	L11423	[10]
Dengue virus	4	DEN-4	325	98.84		1485	U18433	[11]
GB virus C		GBVC	215	96.43	envelope protein (E2)	1006	AF070476	[12]
Hepatitis C virus	1a	HCV-1a	646	100.00	envelope glycoprotein (E1)	585	FJ207922	[13]
Hepatitis C virus	1b	HCV-1b	536	100.00	envelope glycoprotein (E1)	585	HQ113666	
Hepatitis C virus	2a	HCV-2a	77	100.00	Core/E1	1198	L38337	
Hepatitis C virus	2b	HCV-2b	91	100.00	NS5b	563	GQ285070	
Hepatitis C virus	3a	HCV-3a	493	100.00	NS5b	300	JQ303586	

Table S1: continued

**Virus**	**Subtype/ genotype/ serotype**	**Abbrev.**	***N***	**%human**	**Gene/product**	***L***	**Outgroup accession**	**Ref.**
Hepatitis C virus	5a	HCV-5a	246	100.00	envelope glycoprotein (E1)	581	DQ164595	
Hepatitis C virus	6a	HCV-6a	296	100.00	NS5b	922	D84262	
Japanese encephalitis virus	3	JEV	280	5.97	glycoprotein E2	1500	JF706269	[14]
Murray valley encephalitis virus		MVEV	37	2.70	envelope protein (E)	889	U70408	[15]
Tick-borne encephalitis virus	Europe	TBEV-1	124	2.27	envelope glycoprotein (E)	459	GU444281	[7]
Tick-borne encephalitis virus	Far-Eastern	TBEV-2	86	59.38		459	HM468183	
Tick-borne encephalitis virus	Siberian	TBEV-3	298	6.64		459	AY178833	
West Nile virus		WNV	636	10.40	polyprotein	10321	AY532665	[16]
Yellow fever virus		YFV	169	16.67	envelope protein (E)	670	AY640589	[17]
**Orthomyxoviridae**								
Influenza A virus	H1	IAV-H1	1662	100.00	hemagglutinin (HA)	1696	HM567544	[18]
Influenza A virus	H2	IAV-H2	114	100.00		1686	L11129	[19]
Influenza A virus	H3	IAV-H3	7759	100.00		1027	M16737	[20]
Influenza A virus	H5	IAV-H5	367	100.00		1704	GU052518	[21]
Influenza B virus		IBV	3076	73.69	hemagglutinin (HA)	1137	DQ792897	[22]
Influenza C virus		ICV	125	100.00	hemagglutinin-esterase-fusion protein	1968	M11637	[23]
**Paramyxoviridae**								
Human parainfluenza	1	hPIV-1	168	100.00	hemagglutinin-neuraminidase (HN)	1897	M31228	[24]
Human respiratory syncytial virus		RSV	300	100.00	attachment protein (G)	303	M17213	[25]
Measles virus		MeV	501	91.01	hemagglutinin (H)	1854	AY523581	[26]

Table S1: continued

Virus	Subtype/ genotype/ serotype	Abbrev.	*N*	%human	Gene/product	*L*	Outgroup accession	Ref.
Mumps virus		MUV	118	90.24	hemagglutinin-neuraminidase (HN)	1749	GU980052	[26]
**Picornaviridae**								
Coxsackievirus		CA24v	206	100.00	capsid protein (VP1)	915	D90457	[27]
Echovirus	30	E30	1078	99.46	capsid protein (VP1)	879	AJ131523	[28]
Encephalomyocarditis virus		EMCV	43	28.57	RNA-dependent RNA polymerase (3dpol)	312	L22089	[29]
Enterovirus	71	EV71	2861	100.00	capsid protein (VP1)	891	JN874547	[30]
Hepatitis A virus		HAV	251	97.10	capsid protein (VP1)	770	M20273	[31]
Human rhinovirus	A	RhiV-A	190	100.00	polyprotein	6958	NC_001490	[32]
Poliovirus	1,wild	PV-1W	543	100.00	major capsid protein (VP1)	906	AY184220	[33]
**Reoviridae**								
Rotavirus A	1	RotA-1	613	99.18	outer capsid glycoprotein (VP7)	1160	AB018697	[34]
Rotavirus A	12	RotA-12	113	96.77		1160	M58290	
Rotavirus A	2	RotA-2	283	100.00		1160	DQ172849	
Rotavirus A	3	RotA-3	103	100.00		1160	JF805004	
Rotavirus A	4	RotA-4	132	100.00		1160	GU377167	
Rotavirus A	9	RotA-9	282	100.00		1160	AF260959	
Rotavirus C		RotC	58	86.67	outer capsid protein (VP4)	715	M74218	[35]
**Retroviridae**								
Human immunodeficiency virus	A	HIV1-A	172	100.00	negative regulatory factor (nef)	618	CMU11421	[36]
Human immunodeficiency virus	B	HIV1-B	1466	100.00		594	BRRJ045	
Human immunodeficiency virus	C	HIV1-C	623	100.00		612	VI991	

Table S1: continued

Virus	Subtype/ genotype/ serotype	Abbrev.	*N*	%human	Gene/product	*L*	Outgroup accession	Ref.
**Rhabdoviridae**								
Rabies virus	dogs	RabV-1	1016	5.67	nucleoprotein	1349	AF351854	[7]
Rabies virus	bats	RabV-2	717	2.35		1349	EU086201	
Rabies virus	Asian	RabV-3	229	8.90		1349	AB571004	
**Togaviridae**								
Chikungunya virus		ChikV	90	98.57	genome	11851	AF079456	[37]
Crimean-Congo hemorrhagic fever virus		CCHF	189	90.43	nucleocapsid	456	AF434165	[38]
Rubella virus	1	RubV-1	409	99.26	glycoprotein E1	741	DQ975202	[7]
Rubella virus	2	RubV-2	117	95.51		741	EU518611	
**unclassified**								
Hepatitis D virus		HDV	370	100.00	hepatitis delta antigen (HDAg1)	557	AB037949	[7]
Hepatitis E virus		HEV	244	79.58	capsid protein	414	AY535004	[39]

References

[1] Guix S, Caballero S, Villena C, Bartolome R, Latorre C, et al. (2002) Molecular epidemiology of astrovirus infection in Barcelona, Spain. J Clin Microbiol 40: 133–139.

[2] Zou Y, Hu J, Wang ZX, Wang DM, Li MH, et al. (2008) Molecular diversity and phylogeny of Hantaan virus in Guizhou, China: evidence for Guizhou as a radiation center of the present Hantaan virus. J Gen Virol 89: 1987–1997.

[3] Saeed MF, Wang H, Nunes M, Vasconcelos PF, Weaver SC, et al. (2000) Nucleotide sequences and phylogeny of the nucleocapsid gene of Oropouche virus. J Gen Virol 81: 743–748.

[4] Bird BH, Khristova ML, Rollin PE, Ksiazek TG, Nichol ST (2007) Complete genome analysis of 33 ecologically and biologically diverse rift valley fever virus strains reveals widespread virus movement and low genetic diversity due to recent common ancestry. J Virol 81: 2805-16.

[5] Zhang YZ, Dong X, Li X, Ma C, Xiong HP, et al. (2009) Seoul virus and hantavirus disease, Shenyang, People's Republic of China. Emerg Infect Dis 15: 200–206.

[6] Zheng DP, Ando T, Fankhauser RL, Beard RS, Glass RI, et al. (2006) Norovirus classification and proposed strain nomenclature. Virology 346: 312–323.

[7] An arbitrary sequence from a different genotype of the same virus was chosen as an outgroup for the given genotype.

[8] Goncalvez AP, Escalante AA, Pujol FH, Ludert JE, Tovar D, et al. (2002) Diversity and evolution of the envelope gene of dengue virus type 1. Virology 303: 110–119.

[9] Rico-Hesse R, Harrison LM, Salas RA, Tovar D, Nisalak A, et al. (1997) Origins of dengue type 2 viruses associated with increased pathogenicity in the Americas. Virology 230: 244–251.

[10] Messer WB, Gubler DJ, Harris E, Sivananthan K, de Silva AM (2003) Emergence and global spread of a dengue serotype 3, subtype III virus. Emerg Infect Dis 9: 800–809.

[11] Lanciotti RS, Gubler DJ, Trent DW (1997) Molecular evolution and phylogeny of dengue-4 viruses. J Gen Virol 78 (Pt 9): 2279–2284.

[12] Muerhoff AS, Dawson GJ, Desai SM (2006) A previously unrecognized sixth genotype of GB virus C revealed by analysis of 5'-untranslated region sequences. J Med Virol 78: 105–111.

[13] Simmonds P, Bukh J, Combet C, Deleage G, Enomoto N, et al. (2005) Consensus proposals for a unified system of nomenclature of hepatitis C virus genotypes. Hepatology 42: 962–973.

[14] Wang HY, Takasaki T, Fu SH, Sun XH, Zhang HL, et al. (2007) Molecular epidemiological analysis of Japanese encephalitis virus in China. J Gen Virol 88: 885–894.

[15] Gordon AN, Marbach CR, Oakey J, Edmunds G, Condon K, et al. (2012) Confirmed case of encephalitis caused by Murray Valley encephalitis virus infection in a horse. J Vet Diagn Invest 24: 431–436.

[16] Blitvich BJ (2008) Transmission dynamics and changing epidemiology of West Nile virus. Anim Health Res Rev 9: 71–86.

[17] Bryant JE, Holmes EC, Barrett ADT (2007) Out of Africa: a molecular perspective on the introduction of yellow fever virus into the Americas. PLoS Pathog 3: e75.

[18] Influenza H1 sequences were screened for variants associated with the swine-origin H1N1 outbreak of 2009. One of these sequences was used as an outgroup for the remainder of the H1N1 sequences.

[19] Schafer JR, Kawaoka Y, Bean WJ, Suss J, Senne D, et al. (1993) Origin of the pandemic 1957 H2 influenza A virus and the persistence of its possible progenitors in the avian reservoir. Virology 194: 781–788.

[20] Bean WJ, Schell M, Katz J, Kawaoka Y, Naeve C, et al. (1992) Evolution of the H3 influenza virus hemagglutinin from human and nonhuman hosts. J Virol 66: 1129–1138.

[21] Subbarao K, Klimov A, Katz J, Regnery H, Lim W, et al. (1998) Characterization of an avian influenza A (H5N1) virus isolated from a child with a fatal respiratory illness. Science 279: 393–396.

[22] Tsai HP, Wang HC, Kiang D, Huang SW, Kuo PH, et al. (2006) Increasing appearance of reassortant influenza B virus in Taiwan from 2002 to 2005. J Clin Microbiol 44: 2705–2713.

[23] Buonagurio DA, Nakada S, Fitch WM, Palese P (1986) Epidemiology of influenza c virus in man: multiple evolutionary lineages and low rate of change. Virology 153: 12-21.

[24] Seal BS, Crawford JM, Sellers HS, Locke DP, King DJ (2002) Nucleotide sequence analysis of the Newcastle disease virus nucleocapsid protein gene and phylogenetic relationships among the Paramyxoviridae. Virus Res 83: 119–129.

[25] Zlateva KT, Lemey P, Moes E, Vandamme AM, Van Ranst M (2005) Genetic variability and molecular evolution of the human respiratory syncytial virus subgroup B attachment G protein. J Virol 79: 9157–9167.

[26] Pomeroy LW, Bjornstad ON, Holmes EC (2008) The evolutionary and epidemiological dynamics of the paramyxoviridae. J Mol Evol 66: 98–106.

[27] Oberste MS, Maher K, Kilpatrick DR, Pallansch MA (1999) Molecular evolution of the human enteroviruses: correlation of serotype with VP1 sequence and application to picornavirus classification. J Virol 73: 1941–1948.

[28] Kunkel U, Schreier E (2000) Genetic variability within the VP1 coding region of echovirus type 30 isolates. Arch Virol 145: 1455–1464.

[29] An DJ, Jeong W, Jeoung HY, Yoon SH, Kim HJ, et al. (2009) Encephalomyocarditis in Korea: serological survey in pigs and phylogenetic analysis of two historical isolates. Vet Microbiol 137: 37–44.

[30] Wang JR, Tuan YC, Tsai HP, Yan JJ, Liu CC, et al. (2002) Change of major genotype of enterovirus 71 in outbreaks of hand-foot-and-mouth disease in Taiwan between 1998 and 2000. J Clin Microbiol 40: 10–15.

[31] Costa-Mattioli M, Di Napoli A, Ferre V, Billaudel S, Perez-Bercoff R, et al. (2003) Genetic variability of hepatitis A virus. J Gen Virol 84: 3191–3201.

[32] Miller EK, Edwards KM, Weinberg GA, Iwane MK, Griffin MR, et al. (2009) A novel group of rhinoviruses is associated with asthma hospitalizations. J Allergy Clin Immunol 123: 98–104.

[33] Poyry T, Kinnunen L, Hyypia T, Brown B, Horsnell C, et al. (1996) Genetic and phylogenetic clustering of enteroviruses. J Gen Virol 77 (Pt 8): 1699–1717.

[34] A preliminary phylogeny of all rotavirus A sequences was generated using FastTree2 and clusters corresponding to defined genotypes were identified by manual inspection. The alignment was partitioned into genotypes and external outgroup sequences were selected based on their relative position in the phylogeny.

[35] Martella V, Lorusso E, Banyai K, Decaro N, Corrente M, et al. (2008) Identification of a porcine calicivirus related genetically to human sapoviruses. J Clin Microbiol 46: 1907-13.

[36] Kuiken C, Foley B, Leitner T, Apetrei C, Hahn B, et al., editors (2010) HIV Sequence Compendium 2010. Theoretical Biology and Biophysics Group, Los Alamos National Laboratory, NM, LA-UR 10-03684.

[37] Sam IC, Chan YF, Chan SY, Loong SK, Chin HK, et al. (2009) Chikungunya virus of Asian and Central/East African genotypes in Malaysia. J Clin Virol 46: 180–183.

[38] Yashina L, Petrova I, Seregin S, Vyshemirskii O, Lvov D, et al. (2003) Genetic variability of Crimean-Congo haemorrhagic fever virus in Russia and Central Asia. J Gen Virol 84: 1199–1206.

[39] Okamoto H (2007) Genetic variability and evolution of hepatitis E virus. Virus Res 127: 216–228.
